# Dynamic Causal Tractography Analysis of Auditory Descriptive Naming: An Intracranial Study of 106 Patients

**DOI:** 10.1016/j.neuroimage.2025.121319

**Published:** 2025-06-19

**Authors:** Aya Kanno, Ryuzaburo Kochi, Kazuki Sakakura, Yu Kitazawa, Hiroshi Uda, Riyo Ueda, Masaki Sonoda, Min-Hee Lee, Jeong-Won Jeong, Robert Rothermel, Aimee F. Luat, Eishi Asano

**Affiliations:** a Department of Pediatrics, Children’s Hospital of Michigan, Wayne State University, Detroit, MI 48201, USA; b Department of Neurosurgery, Sapporo Medical University, Sapporo, Hokkaido, 0608543, Japan; c Department of Neurosurgery, University of Tohoku, Sendai, Miyagi, 9808575, Japan; d Department of Neurosurgery, University of Tsukuba, Tsukuba, Ibaraki, 3058575, Japan; e Department of Neurosurgery, Rush University Medical Center, Chicago, IL 60612, USA; f Department of Neurology and Stroke Medicine, Yokohama City University Graduate School of Medicine, Yokohama, Kanagawa, 2360004, Japan; g Department of Neurosurgery, Osaka Metropolitan University Graduate School of Medicine, Osaka, 5458585, Japan; h Department of Neurosurgery, National Center Hospital, National Center of Neurology and Psychiatry, Kodaira, Tokyo, 1878551, Japan; i Department of Neurosurgery, Yokohama City University Graduate School of Medicine, Yokohama, Kanagawa, 2360004, Japan; j Department of Neurology, Children’s Hospital of Michigan, Wayne State University, Detroit, MI 48201, USA; k Department of Psychiatry, Children’s Hospital of Michigan, Wayne State University, Detroit, MI 48201, USA; l Department of Pediatrics, Central Michigan University, Mt. Pleasant, Michigan 48858, USA

**Keywords:** language, intracranial electroencephalography (EEG), recording, diffusion tensor imaging (DTI) tractography, broadband high-frequency activity, epilepsy surgery

## Abstract

Humans understand and respond to spoken questions through coordinated activity across distributed cortical networks. However, the causal roles of network engagements alternating across multiple white matter bundles remain understudied at the whole-brain scale. Using intracranial high-gamma activity recorded from 7,792 non-epileptic electrode sites in 106 epilepsy patients who underwent direct cortical stimulation mapping, we constructed an atlas visualizing the millisecond-scale dynamics of functional coactivation and co-inactivation networks during a naming task conducted in response to auditory questions. This atlas, termed the Dynamic Causal Tractography Atlas, identified functional coactivation patterns within specific time windows that were most strongly associated with stimulation-induced language and speech manifestations (p-value range: 2.5 × 10^−5^ to 6.6 × 10^−14^; rho range: +0.54 to +0.82). The atlas revealed that no single intra-hemispheric fasciculus was consistently engaged in all naming stages; instead, each fasciculus supported specific stages, with multiple distinct major fasciculi simultaneously contributing to each stage. Additionally, this atlas identified the specific linguistic stages and fasciculi where handedness effects became evident. Our findings clarify the dynamics and causal roles of alternating, coordinated neural activity through specific fasciculi during auditory descriptive naming, advancing current neurobiological models of speech network organization.

## 1. Introduction

To understand and answer spoken questions, the human brain transforms perceived sounds into meaning, mentally retrieves words relevant to the questions, and converts these words into overt articulation. More specifically, auditory naming-related sensorimotor and cognitive functions include the perception and short-term storage of spoken sounds, semantic-syntactic analyses, lexical retrieval, form encoding, and response initiation and overt articulation (Gathercole et al., 1994; Wise et al., 1999; Hickok and Poeppel, 2000; Levelt, 2001; Liberman and Whalen, 2000; Baddeley, 2003; Liebenthal et al., 2005; Skeide and Friederici, 2016). Current neurobiological models indicate that humans perform this series of functions through coordinated activity via white matter bundles, referred to as fasciculi, predominantly across the left-hemispheric cortical regions (Catani et al., 2005; Dick and Tremblay, 2012; Chang et al., 2015; Gajardo-Vidal et al., 2021). Among these, the left arcuate fasciculus, which dorsally connects the temporal and frontal lobes, is a critical structure for auditory descriptive naming as lesions lead to impairments in semantic-syntactic processing and lexical retrieval (Bernal and Ardila, 2009; Marchina et al., 2011; Wilson et al., 2011; Fridriksson et al., 2013; Griffiths et al., 2013; Gajardo-Vidal et al., 2021; Giampiccolo and Duffau, 2022). Major white matter bundles suggested to support speech processes also include the left superior longitudinal fasciculus (SLF), inferior longitudinal fasciculus (ILF), inferior fronto-occipital fasciculus (IFOF), frontal aslant fasciculus, and uncinate fasciculus (Catani et al., 2005; Mandonnet et al., 2007; Dick and Tremblay, 2012; Friederici and Gierhan, 2013; Chang et al., 2015; Ralph et al., 2017).

Although lesion and electrical stimulation studies have provided insights into the involvement of specific fasciculi in speech (Duffau et al., 2002; Gajardo-Vidal et al., 2021), the dynamics of neural coordination through these fasciculi have been understudied. Investigators suggest that intracranial EEG (iEEG) recording in patients with focal epilepsy can provide a unique opportunity to assess the dynamics of local cortical modulations during overt speech, as well as the neural coordination across cortical regions (Flinker et al., 2015; Anumanchipalli et al., 2019; Woolnough et al., 2021). The advantage of iEEG includes its signal fidelity, which is more than 100 times better than that of scalp EEG and magnetoencephalography (Ball et al., 2009), both of which are inevitably affected by electromyographic artifacts from ocular and temporal muscles during saccadic eye movements and overt articulation (Yuval-Greenberg et al., 2008; Carl et al., 2012). Our previous iEEG study of 13 English-speaking patients with focal epilepsy developed a methodology to assess the dynamics of joint activation and deactivation between pairs of cortical regions during overt naming (Kitazawa et al., 2023). Specifically, we identified pairs of brain regions exhibiting significant, simultaneous, and sustained augmentation of high-gamma amplitude (70–110 Hz), which were directly connected by white matter streamlines derived from MRI tractography of 1,065 healthy individuals in the Human Connectome Project (HCP). Due to the limited sample size, the previous study could only visualize network dynamics across a restricted number of cortical regions and was unable to localize regions showing high-gamma co-attenuation. Furthermore, the study was not designed to evaluate the causal significance of the identified coactivation networks, as it did not correlate coactivation intensity with language-related symptoms elicited by direct electrical stimulation (Kitazawa et al., 2023).

The current study aimed to generate a dynamic causal tractography atlas visualizing functional coactivation networks supporting auditory naming. Using this atlas, we characterized the dynamics of network engagement across 12 major fasciculi during an auditory descriptive naming task and evaluated the independent influences of patient age, handedness, and epilepsy-related factors on these neural dynamics. To assess the causal significance of the identified functional coactivation networks, we correlated coactivation intensity with the probability of given stimulation-induced clinical manifestations, including auditory hallucinations, receptive and expressive aphasia, speech arrest, and facial sensorimotor symptoms (Hamberger, 2007; Tate et al., 2014; Nakai et al., 2017; Lu et al., 2021; Woolnough et al., 2021). We predicted that functional coactivation intensity during auditory stimulus presentation (but not after stimulus offset) would correlate most strongly with stimulation-induced auditory hallucinations, as acoustic processing is expected to begin early in the listening phase and precede semantic analysis (Hickok and Poeppel, 2000; Skeide and Friederici, 2016). Additionally, we predicted that functional coactivation intensity around or after the offset of an auditory question would correlate most strongly with stimulation-induced receptive aphasia. Moreover, we expected this correlation to emerge earlier for receptive aphasia than for expressive aphasia, because listeners must first process the phonemes and comprehend the question before retrieving or generating an appropriate answer (Levelt, 1999; Hickok and Poeppel, 2000; Skeide and Friederici, 2016). Finally, we predicted that the tightest correlation of coactivation intensity with stimulation-induced speech arrest and sensorimotor symptoms would occur around overt response (Chang et al., 2013; Anumanchipalli et al., 2019; Lu et al., 2021). We expected that this analysis would reveal the network organization supporting each stage of language- and speech-related processes necessary for auditory descriptive naming.

Among the 12 major fasciculi investigated in the present study, we predicted that functional coactivation in the left arcuate fasciculus would be the earliest, strongest, and most sustained, given its role in short-term storage of spoken words, semantic-syntactic analysis, and lexical retrieval (Bernal and Ardila, 2009; Marchina et al., 2011; Griffiths et al., 2013; Gajardo-Vidal et al., 2021; Giampiccolo and Duffau, 2022; Janssen et al., 2023; Zhao et al., 2023). Because of its suggested role in semantic analysis or lexical retrieval (Mandonnet et al., 2007; Almairac et al., 2015; Harvey and Schnur, 2015), functional coactivation in the left SLF, ILF, and IFOF would be most prominent between question offset and response onset. Given its involvement in speech initiation (Chernoff et al., 2018; Zhong et al., 2022), functional coactivation in the left frontal aslant fasciculus would be most prominent immediately prior to overt responses. Given the lack of significant symptoms elicited by stimulation or resection (Duffau et al., 2009), the functional coactivation in the left uncinate fasciculus would be modest.

We aimed to enhance the generalizability of our atlas by assessing the impacts of patient age and handedness—independent of epilepsy-related profiles—on task-related neural dynamics. We hypothesized that older age and right-handedness would be associated with greater left-hemispheric dominant functional coactivation during semantic analysis and lexical retrieval periods. Conversely, we hypothesized that younger and left-handed individuals would exhibit more symmetric functional coactivation patterns during these periods. This hypothesis is based on the observation that younger individuals, compared to older ones, display more symmetric language task-related hemodynamic activation on functional MRI (fMRI) (Szaflarski et al., 2006). and show better language function recovery following extensive left-hemispheric damage (Boatman et al., 1999). Additionally, fMRI studies have reported that left-handed healthy individuals, compared to right-handed ones, less frequently exhibit left-hemispheric dominant hemodynamic activation during language tasks (Szaflarski et al., 2002; Mazoyer et al., 2014).

## 2. Materials and methods

### 2.1. Participants

The inclusion criteria for this observational study required participants to be native English speakers with focal seizures who performed an auditory descriptive naming task during extraoperative iEEG recordings at Children’s Hospital of Michigan or Harper University Hospital in Detroit between January 2007 and December 2023. The exclusion criteria were: (i) inability to complete the task, (ii) significant brain malformations affecting the central or lateral sulcus (for example, megalencephaly and perisylvian polymicrogyria), (iii) history of prior resective epilepsy surgery, and (iv) evidence of right-hemispheric language dominance, as indicated by the Wada test results or left-handedness in conjunction with left-hemispheric congenital neocortical lesions. Our eligibility criteria ensured that all study participants had essential language functions in the left hemisphere. We have previously discussed the validity of inferring right-hemispheric language dominance based on left-handedness and left-hemispheric congenital neocortical lesions (Möddel et al., 2009; Sonoda et al., 2022). The Institutional Review Board of Wayne State University approved the current study, and we obtained written informed consent from the patients’ legal guardians and written assent from pediatric patients aged 13 years or older.

### 2.2. Intracranial electrode placement and localization within standard brain template

**Figure 1.**
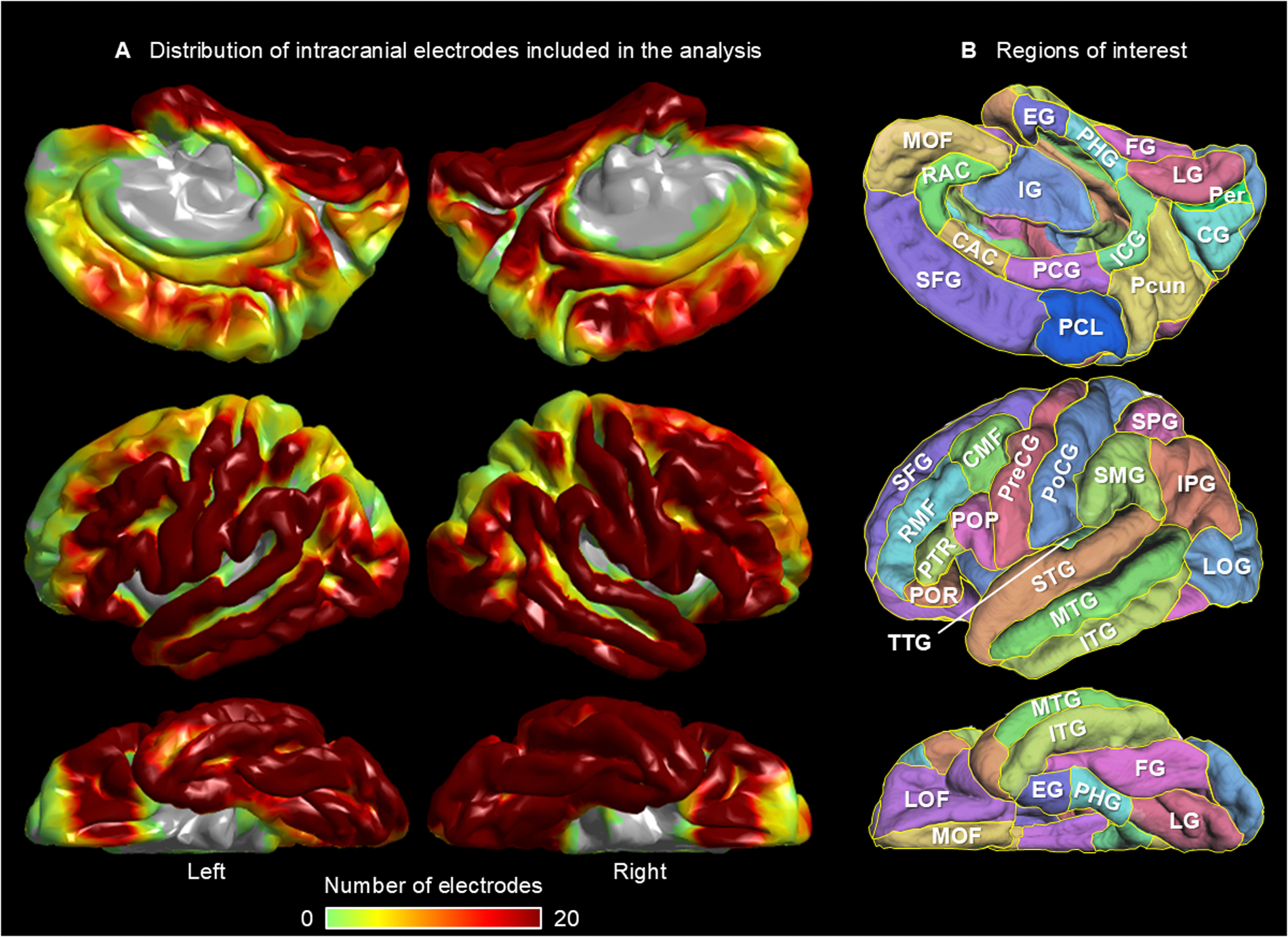
Spatial distribution of intracranial electrode sampling. (A) The figure shows the number of electrodes whose artifact-free, nonepileptic intracranial EEG data were available for measurement of task-related cortical high-gamma dynamics. (B) Regions of interest (ROIs), as defined in the Desikan-Killiany atlas (Desikan et al., 2006), are presented. In Supplementary Table 2, the full names corresponding to the given abbreviations of the ROIs are provided. To generate these images, we used FreeSurfer software (https://surfer.nmr.mgh.harvard.edu/fswiki/CorticalParcellation).

Patients underwent the placement of platinum grid and strip subdural disk electrodes and depth electrodes (10 mm center-to-center; PMT Corporation, Chanhassen, MN, USA) in the intracranial space to localize the boundaries between the epileptogenic zone and eloquent areas. We created a three-dimensional cortical surface image and determined each electrode’s location using preoperative T1 spoiled gradient-recalled MRI, post-implant CT, and the FreeSurfer software package (http://surfer.nmr.mgh.harvard.edu) (Stolk et al., 2018; Sakakura et al., 2023). For group-level analysis, we spatially normalized all electrode locations to the FreeSurfer standard brain space (Desikan et al., 2006; Ghosh et al., 2010; Sakakura et al., 2023). The FreeSurfer script automatically parcellated the cortical gyri and assigned each electrode site a corresponding region of interest (ROI) as defined in the Desikan-Killiany atlas (Figure 1) (Desikan et al., 2006).

### 2.3. Intracranial EEG (iEEG) data acquisition

Following intracranial electrode placement, patients were transferred to the epilepsy monitoring unit, where iEEG was continuously monitored with simultaneous video recording for 3–7 days using the Nihon Kohden Neurofax Digital System (Nihon Kohden America Inc, Foothill Ranch, CA, USA). We acquired iEEG signals at a sampling rate of 1,000 Hz and an amplifier bandpass filter of 0.016–300 Hz. The original reference was the average voltage of the iEEG signals recorded from the amplifier’s fifth and sixth channels (Nariai et al., 2011). The seizure onset zone (SOZ), responsible for generating habitual seizures (Asano et al., 2009), and regions generating interictal spike discharges (Kural et al., 2020) were clinically identified in a prospective manner by a clinical team that included board-certified clinical neurophysiologists and epileptologists. We evaluated the integrity of iEEG signals during the auditory naming task and identified signals affected by electromyographic artifacts (Nagasawa et al., 2011; Uematsu et al., 2013). For further iEEG analysis, we included only artifact-free, nonepileptic electrode sites. Nonepileptic electrode sites were defined as those located outside the SOZ, areas exhibiting interictal spike discharges, and MRI-visible structural lesions (Frauscher et al., 2018; Taylor et al., 2022; Sakakura et al., 2023). Ictal propagation was not used to define non-epileptic electrode sites, as no universally accepted criterion exists for identifying electrodes involved in early propagation. Similarly, the presence or absence of afterdischarges induced by electrical stimulation was not considered when determining whether an electrode site was nonepileptic. Our analytic approach aimed to minimize the effect of epileptiform discharges on task-related high-gamma amplitude measurements (Mercier et al., 2022). Out of 13,648 implanted electrodes, 7,792 were artifact-free, nonepileptic sites. All these 7,792 sites were included for the measurement and visualization of task-related cortical high-gamma dynamics (Figure 1A). Supplementary Table 2 lists the exact number of artifact-free, nonepileptic electrode sites and contributing patients within each ROI. As detailed below, we performed ROI-based iEEG analysis in 54 regions (i.e., 27 in each hemisphere; Figure 1B), each of which contained at least five artifact-free, non-epileptic electrode sites derived from at least three patients.

### 2.4. Auditory descriptive naming task

Patients were awake and comfortably seated on a bed in a room with minimized unwanted noises, and none had a seizure within two hours prior to the task. A series of questions were delivered via a speaker, and we instructed patients to overtly provide an answer (e.g., ‘Bird’ or ‘Plane’) for each question (e.g., ‘What flies in the sky?’); each question began with either ‘what’, ‘where’, ‘when’, or ‘who’ and was designed to elicit one- or two-word answers with nouns (Kambara et al., 2018). This naming task is patient-friendly because no complex task instructions are necessary for children to complete (Nakai et al., 2017). Up to 100 trials were delivered, with the duration of each question ranging from 1.2 to 2.4 seconds (median: 1.8 seconds). Once a patient was confirmed to have verbalized an answer, the examiner delivered the next question. We did not provide interventions to patients during the tasks. At our institution, an investigator was always present in the patient’s private room during cognitive and sensorimotor tasks to directly monitor patient responses and accommodate requests between trials (e.g., offering rest breaks upon request). Consequently, intervals between patient responses and the onset of subsequent questions included in the iEEG analysis varied considerably both within and across patients (mean across patients: 9.6 seconds; standard deviation: 7.6 seconds). Under these experimental conditions, we have previously assessed cognitive task-related iEEG modulations (Johnson et al., 2018; Korzeniewska et al., 2022; Sonoda et al., 2022; Rau et al., 2025). We marked stimulus onset, stimulus offset, and response onset on iEEG signals using sound waves recorded with hands-free microphones (RadioShack 33–3012 Headset Microphone, RadioShack, Miami Beach, FL; Asano and Gotman, 2016). The sound waves were directly recorded via the DC input of the same system used for iEEG acquisition, enabling simultaneous recording of both iEEG signals and sound waves (Asano and Gotman, 2016). We defined the response time as the duration between stimulus offset and response onset. Trials were excluded from further iEEG analysis if a patient failed to provide a relevant answer. The investigator assessed the relevance of each answer. Following Levelt (2001), relevant answers included responses such as ‘bird,’ airplane,’ or ‘animal’ to the question: ‘What flies in the sky?’. Responses such as ‘Can I take a rest?’, ‘Where is my mom?’, or ‘What?’ were considered irrelevant, and such trials were excluded.

### 2.5. Time-frequency analysis

We quantified task-related modulations of high-gamma amplitude, an outstanding summary measure of neural activation due to its strong correlation with increased neural firing rates (Ray et al., 2008; Leszczyński et al., 2020), hemodynamic responses (Scheeringa et al., 2011), and glucose metabolism (Nishida et al., 2008). A meta-analysis of 15 iEEG studies reported that cortical sites showing language task-related high-gamma augmentation were more likely to be classified as part of the language area defined by electrical stimulation mapping (Arya et al., 2018). Our prior iEEG study of 65 patients found that naming task-related augmentation of high-gamma activity at 70–110 Hz, among many spectral frequency bands including slow oscillations, was the best predictor of postoperative language outcomes (Sonoda et al., 2022).

This time-frequency analysis was conducted using a bipolar montage approach, which reduces contamination from volume conduction by measuring potential differences between adjacent electrode pairs. For cortical electrode sites bordered by two or more neighboring electrode pairs, the high-gamma amplitude was determined by averaging the values derived from these pairs. For example, the high-gamma amplitude at cortical electrode site A2 on a depth electrode was calculated as the average of measurements from electrode pairs A1–A2 and A2–A3 (Johnson et al., 2023; Kitazawa et al., 2025). Depth electrodes positioned solely within white matter were not included in the analysis.

We transformed iEEG signals into 10-ms/5-Hz time–frequency bins using the complex demodulation method incorporated in the BESA EEG Software (BESA GmbH, Gräfelfing, Germany) (Papp and Ktonas, 1977; Hoechstetter et al., 2004). This process involved multiplying the time-domain iEEG signal with a complex exponential, followed by a band-pass filter. Because a Gaussian-shaped low-pass finite impulse response filter was employed, this complex demodulation method is equivalent to a Gabor transformation. The time–frequency resolution for high-gamma measurement was ±15.8 ms and ±7.1 Hz (defined as the 50% power drop of the finite impulse response filter). We aligned the iEEG traces to stimulus onset, stimulus offset, and response onset to determine the percent change in high-gamma amplitude at 70–110 Hz compared to the average value during the baseline period, defined as the 400-ms interval between 200 and 600 ms prior to stimulus onset. At all artifact-free, nonepileptic electrode sites, we quantified the percent change in high-gamma amplitude during the following periods: between −200 and +500 ms relative to stimulus onset; between −500 and +500 ms relative to stimulus offset; between −500 and +500 ms relative to response onset. Here, ‘amplitude’ is a measure proportional to the square root of ‘power’. We then visualized the percentage change in high-gamma amplitude on a FreeSurfer standard brain template, using interpolation within a 10 mm radius from the electrode center (Sakakura et al., 2023). Thereby, high-gamma amplitudes were first averaged across trials within each patient and subsequently averaged across patients. We used MATLAB R2023 software for visualization purposes (MathWorks, Natick, MA, USA).

In each ROI, we declared a significant augmentation of high-gamma amplitude when the lower limit of the 99.99% confidence interval (CI) of the mean across electrode sites exceeded zero for a duration of 50 ms or more. Similarly, we declared a significant attenuation when the upper limit of the 99.99% CI consistently fell below zero for at least 50 ms. Using a 99.99% CI corresponds to a Bonferroni correction for 500 repeated comparisons, and a 50-ms interval would include a minimum of three high-gamma oscillatory cycles. A duration of 50 ms was selected to include at least three cycles of high-gamma oscillations, providing a physiologically meaningful criterion. Even simple sensory stimuli, such as somatosensory or visual flash stimulation—which involve minimal cognitive processing—typically induce high-gamma augmentation lasting at least 50 ms in both lower- and higher-order perceptual areas (Fukuda et al., 2008; Avanzini et al., 2016; Nakai et al., 2018). Therefore, we anticipated that neural communication underlying cognitive functions between distinct cortical gyri would require neural activation lasting at least 50 ms.

A 99.99% one-sided test corresponds to an alpha (false-positive probability) of 0.0001. Under the null hypothesis, the chance probability of observing a significant high-gamma increase in any given time bin is therefore 0.0001. If we assume temporal independence (no correlation) between adjacent time bins, the chance probability of observing significant high-gamma augmentation across 5 consecutive time bins in each ROI is (0.0001)^5^ × (250 − 5 + 1) = 2.46 × 10^−18^, an exceedingly low probability. The chance probability of observing significant high-gamma attenuation across 5 consecutive time bins is likewise 2.46 × 10^−18^.

Since high-gamma amplitudes in adjacent time bins are likely temporally autocorrelated, we also considered scenarios accounting for temporal dependence. The impact of autocorrelation on functional connectivity estimates is extensively documented in the fMRI literature (e.g., Arbabshirani et al., 2014; Afyouni et al., 2019; Shinn et al., 2023). However, we were unable to identify previous iEEG studies quantifying the conditional probability of observing significant high-gamma augmentation immediately following another significant augmentation during language tasks. Animal studies using cortical slices suggest that neural activation probability (e.g., firing rate) can transiently increase up to ten-fold immediately following local bursts of excitatory activity (Ikegaya et al., 2004; Harris, 2005; Sridharan et al., 2011). If we conservatively assume an even higher conditional probability (e.g., a 100-fold increase, resulting in a conditional probability of 0.01) for observing significant high-gamma augmentation in a time bin immediately following a significant augmentation, the resulting chance probability of detecting significant high-gamma augmentation across five consecutive bins at each ROI would be calculated as (0.0001) × (0.01)^4^ × (250 − 5 + 1) = 2.46 × 10^−10^. Even under an extreme scenario of very high temporal dependence (e.g., conditional probability increased 1, 000-fold to 0.1), this chance probability rises to (0.0001) × (0.1)^4^ × (250 − 5 + 1) = 2.46 × 10^−6^, which remains acceptably low.

### 2.6. Visualization of white matter streamlines on tractography

Our aims included generating new white matter streamline templates and providing these templates as open-source material. iEEG investigators commonly use standard brain templates, such as the Montreal Neurological Institute (MNI) standard space image (Jenkinson et al., 2012) and the Desikan-Killiany atlas (Desikan et al., 2006), to visualize neural dynamics at a group level. The iEEG research guidelines do not mandate investigators to create an anatomical brain template derived from study participants (Mercier et al., 2022). Creating an average brain template involves labor-intensive and time-consuming processes, such as manually inspecting and correcting artifactual signals and topological defects on anatomical MRI. Additionally, standard anatomical brain templates are generally assumed to represent the average brain anatomy of patients with reasonable spatial accuracy. Our previous iEEG-tractography study of 37 children with focal epilepsy, aged 5 to 20 years, suggests that the anatomical courses of white matter pathways are quite similar between patients and healthy individuals (Sonoda et al., 2021). We found that the lengths of white matter streamlines in individual patients’ tractography were highly correlated (Pearson correlation coefficient of 0.8) with those measured in 1,065 healthy individuals participating in the HCP. Thus, in the present study, we used this HCP tractography dataset to generate white matter streamline templates (http://brain.labsolver.org/diffusion-mri-templates/hcp-842-hcp-1021) (Yeh et al., 2018). Using the DSI Studio script (http://dsi-studio.labsolver.org/) within the MNI standard space, we constructed, validated, and visualized the streamline connecting each pair of cortical ROIs with the shortest length among those detected using the specified parameters. We have provided the details of the fiber tracking and visual validation procedures in Supplementary Figures 1 and 2. Board-certified neurosurgeons (A.K. and R.K.) classified each of the validated white matter streamlines into one of the following 13 categories: (i) arcuate fasciculus, (ii) SLF, (iii) middle longitudinal fasciculus (MLF), (iv) ILF, (v) IFOF, (vi) frontal aslant fasciculus, (vii) parietal aslant fasciculus, (viii) uncinate fasciculus, (ix) cingulum fasciculus, (x) extreme capsule, (xi) intra-hemispheric U-fibers (i.e., other than those listed above), (xii) callosal fibers, or (xiii) anterior commissural fibers. Supplementary Figures 2 and 3 provide the criteria used to define these fasciculi and visualizes the spatial characteristics of white matter streamlines classified into specific categories.

### 2.7. Definition and visualization of task-related functional coactivation networks

To characterize the functional coactivation network (Kitazawa et al., 2023; 2025; Ono et al., 2023; Ueda et al., 2024), we subsequently identified time periods during which high-gamma amplitude was simultaneously augmented across pairs of ROIs. Functional coactivation between two ROIs was defined by two criteria: (i) both ROIs must exhibit significant, simultaneous high-gamma augmentation lasting at least five consecutive 10-ms time bins, and (ii) the ROIs must be directly connected by a white matter streamline identified on HCP diffusion-weighted imaging (DWI) tractography. A unique aspect of our definition is the incorporation of millisecond-scale high-gamma co-augmentation (rather than coherent iEEG fluctuations over extended periods), combined with identification of white matter pathways between cortical ROIs.

To justify our analytic approach, we evaluated the chance probability that observed high-gamma co-augmentation at two ROIs. Under the null hypothesis, the chance probability of observing a significant high-gamma increase at an individual ROI during any single time bin is 0.0001. Therefore, the chance probability of observing significant high-gamma augmentation simultaneously at a pair of ROIs during a single time bin is (0.0001 × 0.0001) = 10^−8^. Assuming an extremely high temporal autocorrelation of high-gamma amplitudes across adjacent time bins, such that the conditional probability of observing augmentation increases 1,000-fold immediately following a significant augmentation, the chance probability of detecting significant high-gamma augmentation across five consecutive time bins at the same ROI pair is calculated as: 10^−8^ × (0.1)^4^ = 10^−12^. Given an analysis window consisting of 250 time bins and 54 ROIs (generating 1,431 ROI pairs), the overall chance probability of observing significant and simultaneous high-gamma co-augmentation lasting for at least five consecutive bins at least at an ROI pair is 10^−12^ × (250 − 5 + 1) × 1,431 = 3.52 × 10^−7^. The chance probability of observing simultaneous high-gamma co-attenuation is likewise 3.52 × 10^−7^. These findings suggest that the observed coactivation/co-inactivation networks are unlikely to be explained by chance.

We visualized the temporal profiles and intensity of functional coactivation and co-inactivation across 10 intra-hemispheric fasciculi, local U-fibers, and two inter-hemispheric white matter pathways mentioned above. We calculated the onset (ms), duration (ms) and maximum intensity (%) of functional coactivation for each white matter pathway. The intensity of functional coactivation between ROI_1_ and ROI_2_ at a given time bin was defined as the square root of [high-gamma amplitude % change at ROI_1_ × high-gamma amplitude % change at ROI_2_]. If no streamline directly connected ROI_1_ and ROI_2_, the intensity of functional coactivation between these regions was considered zero.

### 2.8. Electrical stimulation mapping

As part of the routine presurgical evaluation, we performed electrical stimulation mapping (Nakai et al., 2017). Before initiating electrical stimulation mapping, the neuropsychologist conducted a practice session to confirm that the patient could readily answer a given auditory question during the baseline period. Questions that could not be answered during this period were excluded from electrical stimulation mapping. We delivered a pulse train of repetitive electrical stimuli to adjacent electrode pairs, with a stimulus frequency of 50 Hz, a pulse duration of 0.3 ms, and a train duration of up to 5 seconds. The stimulus intensity was initially set at 3 mA and increased stepwise up to 9 mA until investigators observed a clinical response or after-discharge. During each trial, patients were asked to answer auditory questions beginning with ‘what’, ‘when’, ‘where’, or ‘who’. Electrode sites where stimulation reproducibly elicited clinical symptoms without inducing afterdischarges were prospectively identified and assessed at bedside by at least two investigators, including a neuropsychologist (R.R.), both blinded to task-related high-gamma dynamics. If a patient failed to verbalize an answer during stimulation, the reason was clarified through direct inquiry. The neuropsychologist considered this information when determining which impaired language process was responsible for a given clinical symptom. For example, if a patient stated, ‘I understood your question—you asked me what flies in the sky—but I could not tell you the answer,’ the neuropsychologist would exclude receptive aphasia. Additional tasks, such as humming, syllable repetition, counting, and reciting the alphabet, were performed to further delineate the specific functional role of each cortical site where stimulation elicited clinical symptoms.

We included the following as language- or speech-related symptoms:

- Auditory hallucination, defined as the perception of sounds at various pitches or alterations in the neuropsychologist’s voice pitch.
- Receptive aphasia, defined as an inability to understand the question despite awareness that a question had been asked.
- Expressive aphasia, defined as dysnomia, paraphasic or nonsensical speech, or an inability to provide a relevant answer within the 5-second stimulation interval, provided the patient clearly understood the question and was able to initiate vocalization (i.e., speech initiation occurred, but speech content was abnormal or incorrect).
- Speech arrest, defined specifically as a complete inability to initiate or continue vocalization during stimulation, without evidence of forced jerking or involuntary motor movements of the tongue or lips.
- Face sensorimotor symptoms, defined as involuntary facial movement or sensation, with or without overt dysarthria.

Paraphasic or nonsensical speech responses were classified as expressive aphasia if the patient successfully repeated the question immediately after stimulation but classified as receptive aphasia if the patient failed to do so. Brief hesitation was disregarded if the patient answered before stimulation offset. However, if the patient responded only after stimulation offset and this delay could not be attributed to speech arrest (defined as complete interruption of vocalization; e.g., inability to repeat syllables), the symptom was classified as expressive aphasia.

### 2.9. Assessment of patient demographics and epilepsy characteristics

We employed a mixed model analysis to determine the impact of patient demographics and epilepsy-related profiles on response time. We included the following variables in the mixed model analysis: (i) square root of patient age (√years), (ii) handedness (1 if left-handed; 0 otherwise), (iii) left-hemispheric epileptogenicity (1 if surgical intervention or SOZ involved the left hemisphere; 0 otherwise), (iv) an MRI-visible lesion (1 if present; 0 if absent), and (v) the number of antiseizure medications taken immediately prior to the iEEG recording. We incorporated the square root of age in the mixed model, assuming that the developmental changes in naming behaviors and neural systems would be greater in younger individuals. We interpreted the need for multiple antiseizure medications as indicative of greater seizure-related cognitive burdens (Kwan and Brodie, 2001; Kuroda et al., 2021). The median response time was treated as the dependent variable, while patient was included as a random factor, allowing the intercept to vary across patients. We used SPSS Statistics 28 (IBM Corp., Chicago, IL, USA) for statistical analyses. All reported p-values are two-sided, and we considered a p-value of <0.05 significant.

Likewise, we employed a mixed model analysis to determine the impact of aforementioned patient demographics, epilepsy-related profiles, and response time on high-gamma amplitude (% change compared to baseline) at each ROI. The dependent variable was the median at each electrode site during each 100-ms epoch within the 2,500-ms period between stimulus onset and 500-ms after the response onset. Given the repeated analyses across 54 ROIs and 25 100-ms epochs, we considered a Bonferroni-corrected p-value of <0.05 as significant.

### 2.10. Statistical assessment of the relationship between functional coactivation and stimulation-induced manifestations

We assessed the association between the intensity of functional coactivation during specific time windows and language- or speech-related functions identified through electrical stimulation mapping. Using data from 54 ROIs, we applied Spearman’s rank correlation tests to assess the correlation between the mean intensity of functional coactivation (%) and the probability (%) of eliciting specific stimulation-induced symptoms at each ROI. Each ROI exhibited distinct coactivation intensities at each 10-ms time bin and associated symptom probabilities, ensuring sufficient variability for this analysis (N = 54).

The intensity of functional coactivation between a pair of ROIs (${ROI}_{i}$ and ${ROI}_{j}$)at a given time bin was defined as:

$$\sqrt{\text{(high-gamma}\;\text{amplitude}\;\left.\%\;\text{change}\;\text{at}\;{ROI}_{i}\right)\times \left(\text{high-gamma}\;\text{amplitude}\;\%\;\text{change}\;\text{at}\;{ROI}_{j}\right)}$$

Consequently, the mean intensity of functional coactivation at a given ROI (${ROI}_{i}$) during a specific time bin was calculated by averaging its functional coactivation intensities with all other ROIs:

$$\sum_{j=1,j\neq i}^{54}\sqrt{\;(\text{high-gamma}\;\text{amplitude}\;\%\;\text{change}\;\text{at}\;{ROI}_{i})\times (\text{high-gamma}\;\text{amplitude}\;\%\;\text{change}\;\text{at}\;{ROI}_{j})}$$

The probability of eliciting specific stimulation-induced symptoms, defined as the proportion of electrode sites showing clinical symptoms among all tested sites, was computed for each of the 54 ROIs. Using these probabilities and the functional coactivation intensity at each ROI calculated in 10-ms time bins, Spearman’s rank correlation tests identified time periods during which coactivation intensity significantly correlated with symptom probability. A Spearman’s rho greater than zero indicates a link between task-related functional coactivation in a specific 10-ms time bin and a specific language-related function indicated by electrical stimulation mapping. Given that we performed Spearman’s rank test for 250 time bins (i.e., 2,500-ms time periods), a Bonferroni-corrected p-value of <0.05 was considered significant.

## 3. Results

### 3.1. Patient profiles and analyzed electrode sites

A total of 106 patients with drug-resistant focal epilepsy, who met the inclusion and exclusion criteria, were included in the study (age range: 4–41 years; 49 females; 8 left-handed; 52 MRI-nonlesional cases). Detailed patient demographic information is provided in Supplementary Table 1. Out of 13,648 implanted electrodes, 7,792 were artifact-free, nonepileptic sites. All these 7,792 sites were included for the measurement and visualization of task-related cortical high-gamma dynamics (Figure 1A). Supplementary Table 2 lists the exact number of artifact-free, nonepileptic electrode sites and contributing patients within each ROI.

### 3.2. Impact of patient demographics and epilepsy-related profiles on response time

Univariate regression analysis confirmed that there was no excessive multicollinearity across √age, handedness, left-hemispheric epileptogenicity, MRI-visible lesion, and the number of antiseizure medications (range of Coefficient of Determination R^2^: 0.0001 to 0.041; range of uncorrected p-values: 0.039 to 0.91). The mixed model analysis, incorporating all five variables as fixed effect factors, revealed that increased √age was associated with a reduction in the median response time (mixed model estimate: −0.246 [95% CI: −0.40 to −0.088]; t-value: −3.10; p-value: 0.003), independent of the other fixed effect variables. This finding indicates that each additional √year (e.g., from 9 to 16 years) was associated with a 246-ms reduction in response time. None of the other variables showed a significant association with median response time (range of p-values: 0.15 to 0.78).

### 3.3. Impact of patient demographics, epilepsy-related profiles, and response time on neural dynamics

The mixed model analysis indicated that longer response time was associated with smaller high-gamma amplitude in the left precentral gyrus after stimulus offset. In other words, individuals with shorter response times showed greater engagement of the left-hemispheric motor cortex, occurring 200–500 ms after stimulus offset. Specifically, each 1-second prolongation of response time was associated with reduced high-gamma amplitude at 400–500 ms after stimulus offset by 9.70% (95%CI: −13.12% to −6.28%; uncorrected p-value: 1.4 × 10^−6^; t-value: −5.75; DF: 37.10), at 300–400 ms after stimulus offset by 8.95% (95%CI: −12.20% to −5.71%; uncorrected p-value: 2.3 × 10^−6^; t-value: −5.60; DF: 36.79), and at 200–300 ms after stimulus offset by 8.09% (95%CI: −11.38% to −4.80%; uncorrected p-value: 1.5 × 10^−5^; t-value: −4.98; DF: 36.44).

The mixed-model analysis also indicated that left-handedness was associated with greater high-gamma amplitude in two right-hemispheric ROIs during the 200–500 ms interval preceding response onset. In other words, compared to right-handed individuals, left-handed individuals showed milder high-gamma attenuation in these right-hemispheric ROIs several hundred ms prior to overt responses. Specifically, left handedness increased right entorhinal high-gamma amplitude at 400–500 ms before response by 9.32% (95%CI: 5.39% to 13.25%; uncorrected p-value: 1.3 × 10^−5^; t-value: 4.74; DF: 62.00), at 300–400 ms before response by 11.68% (95%CI: 7.80% to 15.60%; uncorrected p-value: 3.0 × 10^−6^; t-value: 6.18; DF: 22.36), and at 200–300 ms before response by 12.60% (95%CI: 7.50% to 17.70%; uncorrected p-value: 3.6 × 10^−5^; t-value: 5.12; DF: 22.76). Left handedness likewise increased right supramarginal high-gamma amplitude at 400–500 ms before response by 14.39% (95%CI: 8.57% to 20.22%; uncorrected p-value: 1.2 × 10^−5^; t-value: 4.99; DF: 40.05). These observations indicate that functional co-inactivation in the right parietal aslant fasciculus between the entorhinal and supramarginal gyri, occurring 400–500 ms before response, was 11.5% milder in left-handed individuals compared to right-handed ones. No other ROIs showed significant effects in the mixed-model analysis.

### 3.4. Summarized view of functional coactivation and co-inactivation during auditory descriptive naming

**Figure 2.**
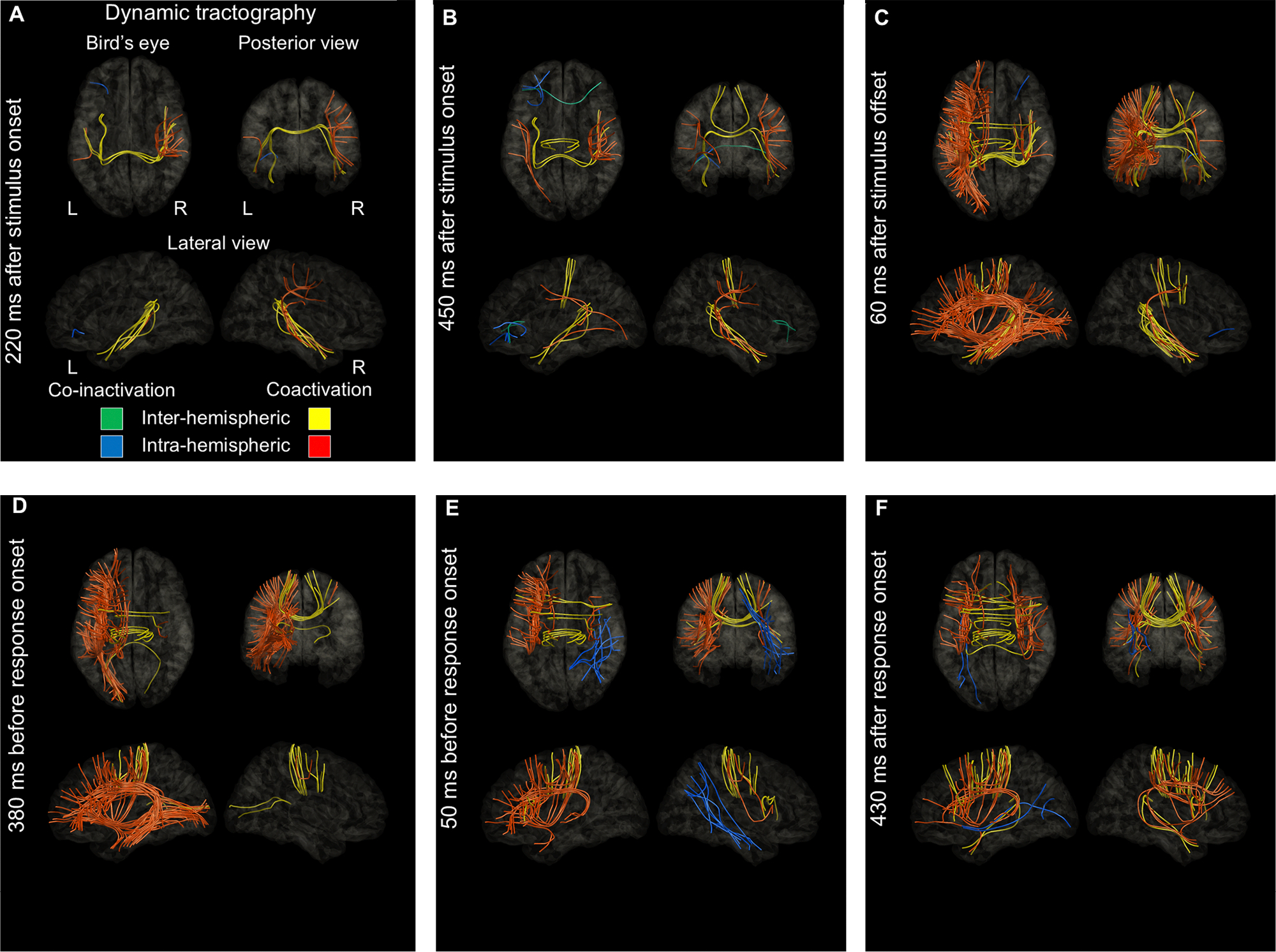
Functional coactivation and co-inactivation during auditory naming. Snapshots illustrate the dynamics of functional coactivation and co-inactivation occurring during an auditory naming task. Orange and yellow streamlines: intra-hemispheric and inter-hemispheric functional coactivation. Blue and green streamlines: intra-hemispheric and inter-hemispheric functional co-inactivation. (A) 220 ms after stimulus onset (association with auditory hallucination). (B) 450 ms after stimulus onset. (C) 60 ms after stimulus offset (association with receptive aphasia). (D) 380 ms before response onset (association with expressive aphasia). (E) 50 ms before response onset (association with speech arrest). (F) 430 ms after response onset (association with face sensorimotor symptoms). For a comprehensive overview of the network dynamics, please refer to Supplementary Video 1. L: left. R: right.

**Figure 3.**
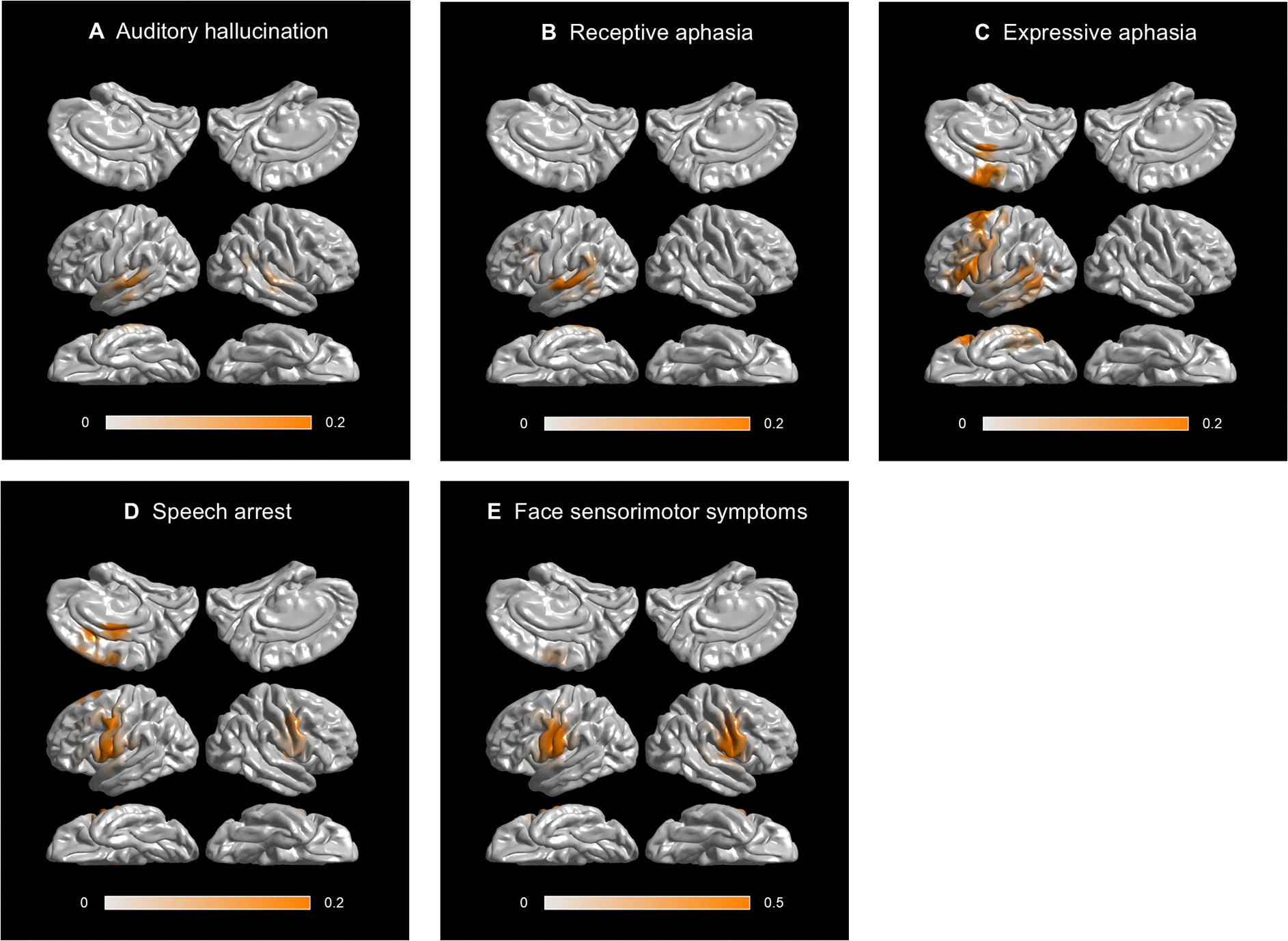
Probability map of stimulation-induced language-related symptoms. (A) Auditory hallucinations. (B) Receptive aphasia. (C) Expressive aphasia. (D) Speech arrest. (E) Face sensorimotor symptoms.

**Figure 4.**
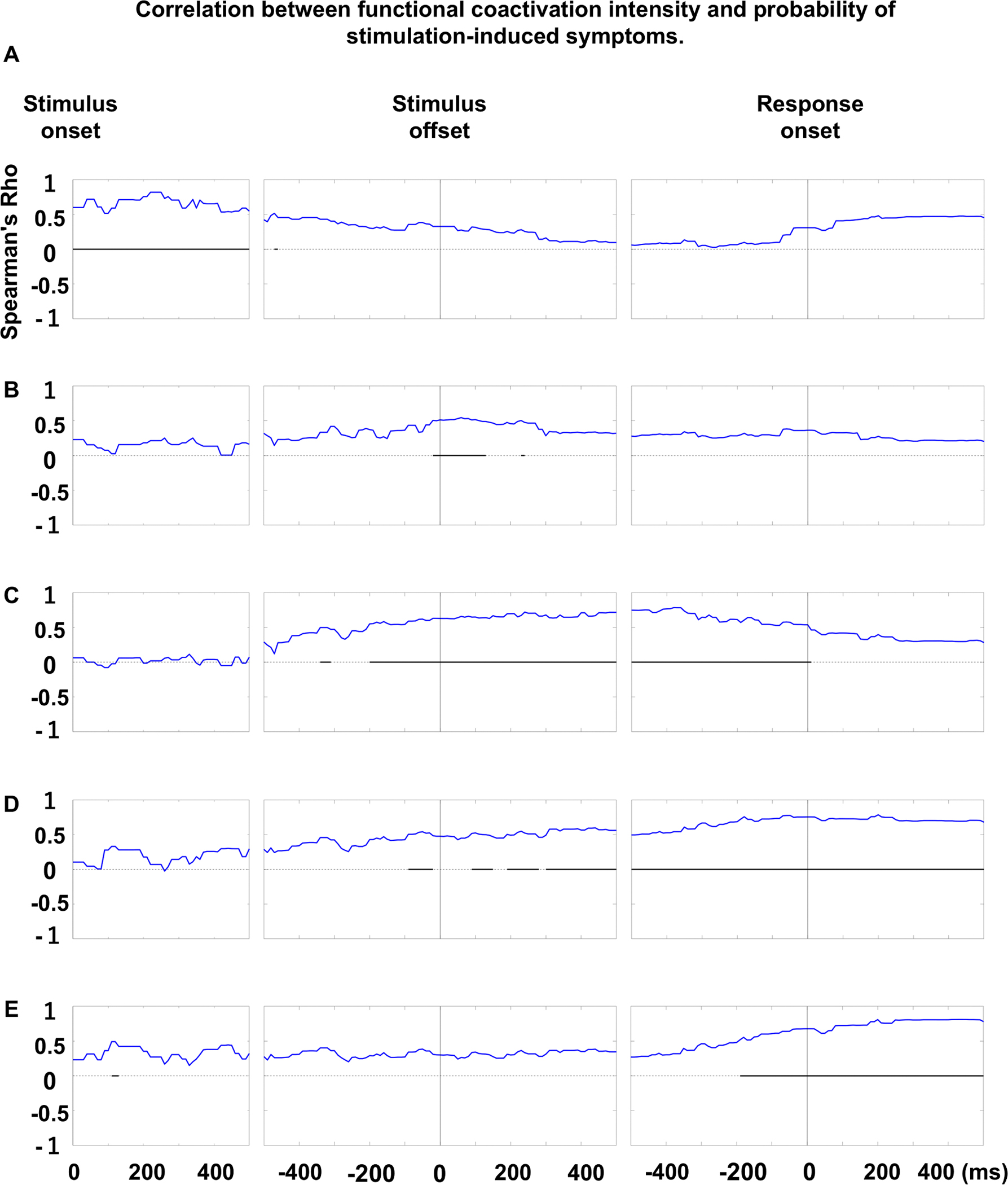
Association between functional coactivation and stimulation-induced symptoms. Each plot displays Spearman’s rho, indicating the strength of the correlation between the probability of a given stimulation-induced manifestation and the mean functional coactivation intensity over time bins. (A) Auditory hallucination. (B) Receptive aphasia. (C) Expressive aphasia. (D) Speech arrest. (E) Facial sensorimotor symptoms. Horizontal bars indicate significant correlations based on a Bonferroni-corrected p-value of less than 0.05.

**Figure 5.**
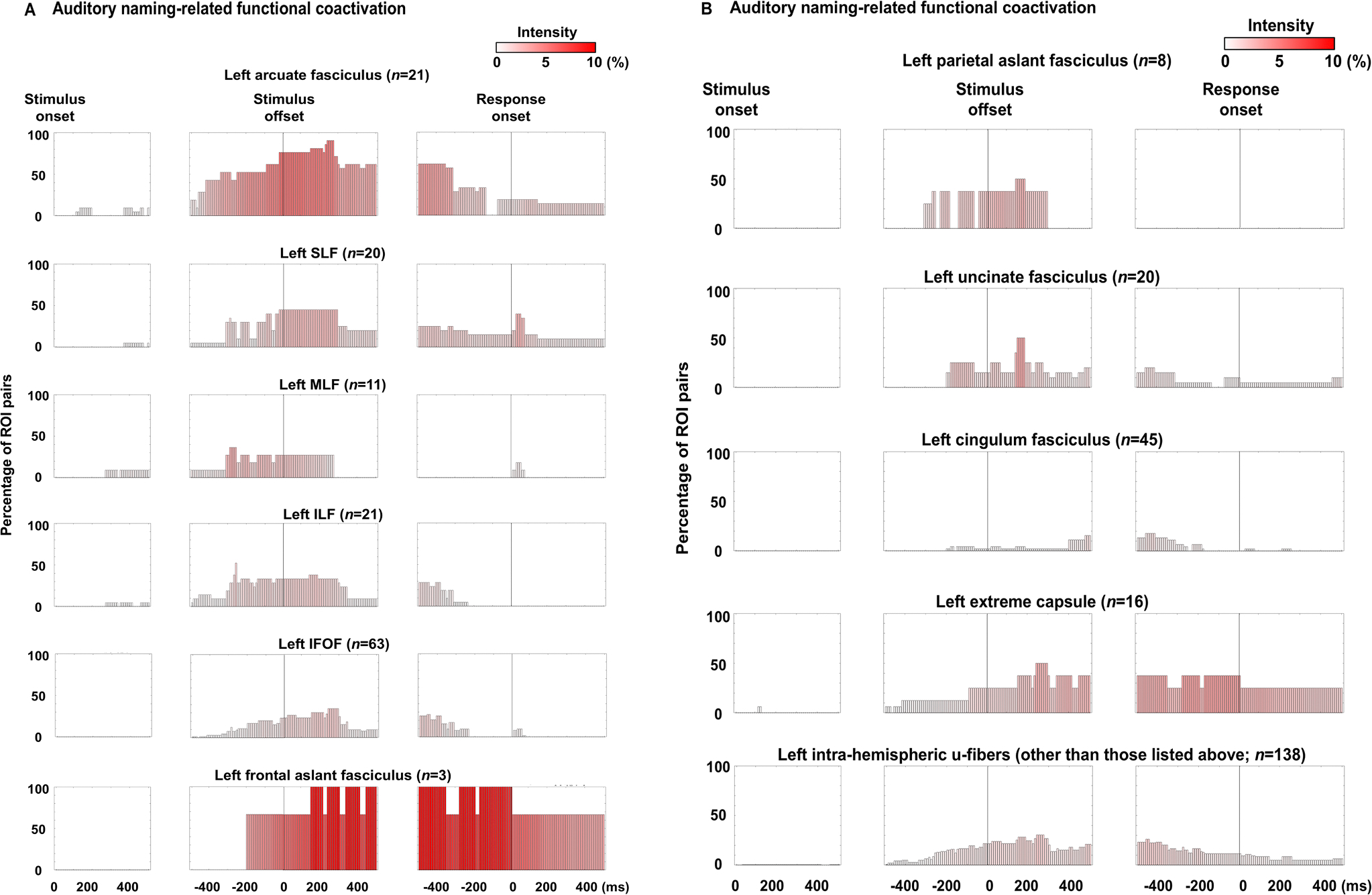

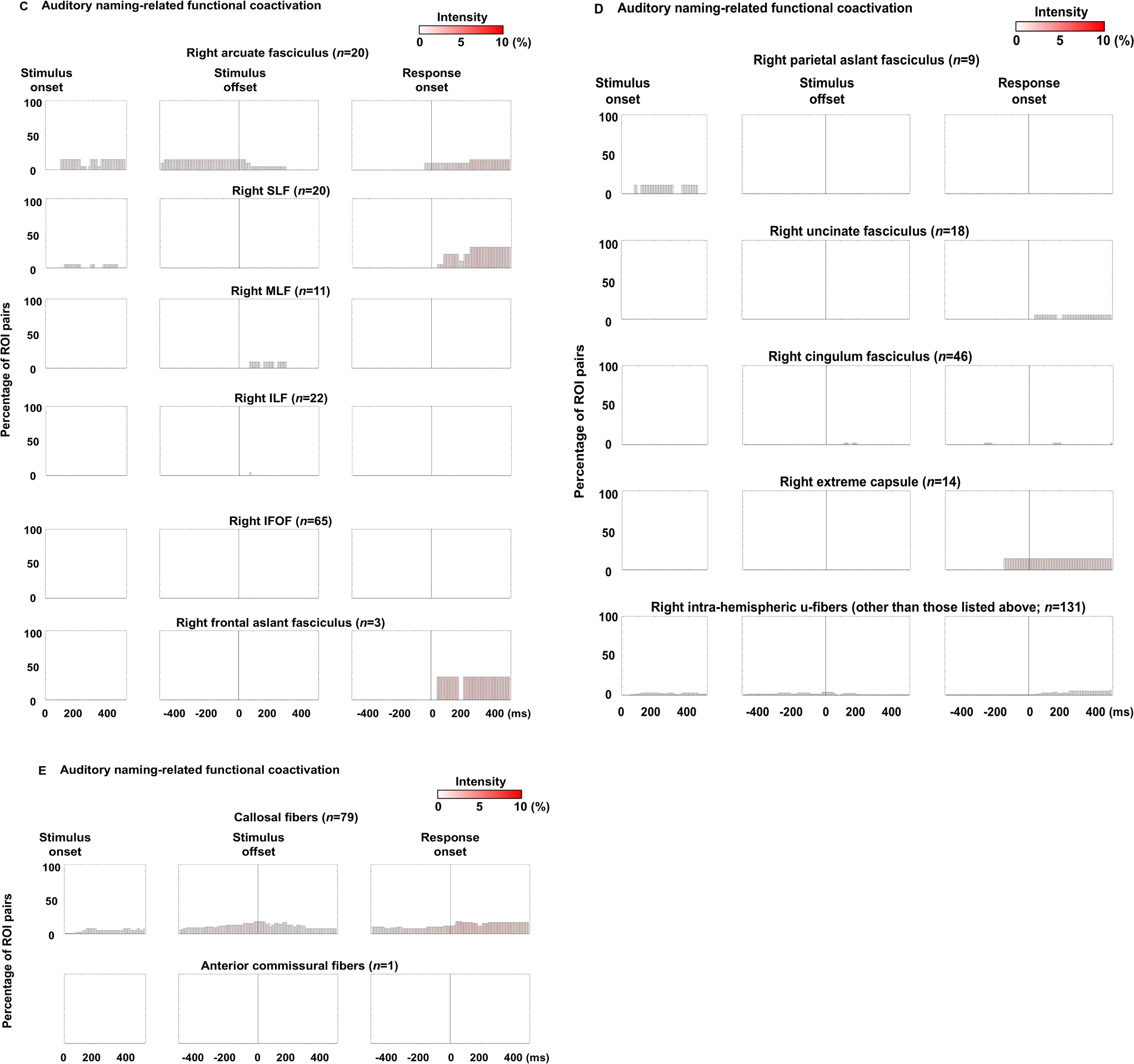
Dynamics of functional coactivation through each fasciculus. The bar height represents the proportion of functional coactivation within each fasciculus for a given time bin, while the bar color reflects the average intensity of functional coactivation within each fasciculus. (A, B) Left intra-hemispheric pathways. (C, D) Right intra-hemispheric pathways. (E) Inter-hemispheric pathways. Supplementary Figure 5 presents the dynamics of functional co-inactivation.

Figure 2 and Supplementary Video 1 provide a comprehensive overview of the dynamics of functional coactivation and co-inactivation, as well as cortical high-gamma modulations (Supplementary Figure 4). Figure 3 illustrates probability maps for stimulation-induced language-related manifestations, and Figure 4 identifies the specific time bins during which the intensity of functional coactivation significantly correlated with the likelihood of each stimulation-induced manifestation. In addition, we visually summarize the duration (ms), extent (% of ROI pairs showing significant coactivation), and intensity (%) of functional coactivation (Figure 5) and co-inactivation (Supplementary Figure 5) within each fasciculus.

For interested readers, Supplementary Figure 6 presents temporal changes in group-level high-gamma amplitudes at given ROIs using a leave-one-out approach, demonstrating that the observed high-gamma modulation reflects a consistent group-level pattern rather than being driven by any single individual. Supplementary Figure 7 shows temporal changes in mean high-gamma amplitude at each ROI, including 95% confidence interval bars. Supplementary Figure 8 illustrates temporal changes in the number of ROIs exhibiting significant high-gamma augmentation and attenuation, effectively indicating when greater numbers of ROIs were engaged or disengaged during the auditory naming task.

Upon hearing auditory stimuli, inter-hemispheric functional coactivation was noted between the superior temporal gyrus (STG) via the corpus callosum, while intra-hemispheric functional coactivation was noted between the STG and precentral gyrus through the arcuate fasciculus in each hemisphere (Figure 2A). The STG was also involved in functional coactivation through local U-fibers in each hemisphere. Conversely, the left IFG was involved in intra- and inter-hemispheric functional co-inactivation through local frontal U-fibers and callosal fibers, while patients listened to auditory questions (Figure 2B). Subsequently, as stimulus offset approached, left intra-hemispheric functional coactivation became more evident through multiple fasciculi. After stimulus offset, functional coactivation became extensive in the left hemisphere (Figures 2C and 2D), whereas functional co-inactivation became evident in the right hemisphere (Supplementary Figures 5C and 5D). Around 100 ms prior to response onset, functional coactivation within the left frontal lobe regions persisted, while coactivation between the left frontal and temporal or occipital regions, including the arcuate and IFOF, diminished. Additionally, inter-hemispheric functional coactivation between the precentral gyri was observed through the corpus callosum immediately before and during responses (Figure 2E). During overt responses, intra-hemispheric functional coactivation between the STG and precentral gyrus persisted via the arcuate fasciculus in both hemispheres. Inter-hemispheric functional coactivation between the Rolandic regions and between the STG also persisted through the corpus callosum (Figure 2F).

### 3.5. Electrical stimulation mapping

Stimulation-induced auditory hallucination, receptive aphasia, expressive aphasia, speech arrest, and face sensorimotor symptoms were observed at 50, 58, 146, 143, 518 sites, respectively. Auditory hallucination sites were primarily localized in the bilateral STG, whereas face sensorimotor sites were symmetrically distributed in the bilateral Rolandic cortices (Figures 3A and 3E). In contrast, receptive aphasia, expressive aphasia, and speech arrest sites were predominantly localized in the left hemisphere (Figures 3B–3D). Spearman’s rank correlation test did not reveal a correlation between patient age and the minimum stimulus intensity required to elicit a given clinical symptom (Bonferroni-corrected p-values: >0.05; detailed results are provided in the Supplementary Document).

### 3.6. Functional coactivation associated with stimulation-induced auditory hallucinations

A significant association was found between increased probability of stimulation-induced auditory hallucinations and increased functional coactivation intensity upon auditory stimulus presentation, with the strongest correlation occurring at 220–250 ms after stimulus onset (rho: +0.82; uncorrected p-value: 6.6 × 10^−14^; t-value: +10.2; Figure 4A). Figure 2A presents the spatial characteristics of intra- and inter-hemispheric functional coactivation 220 ms after stimulus onset. Around this time, the callosal fiber connecting the left and right STG, as well as the arcuate fasciculi linking the STG and precentral gyrus, were among the major fasciculi exhibiting functional coactivation.

### 3.7. Functional coactivation associated with stimulation-induced receptive aphasia

Similarly, a significant association was noted between increased probability of stimulation-induced receptive aphasia and increased functional coactivation intensity during a brief period around stimulus offset (Figure 4B), and the strength of association was maximized at 60 ms after stimulus offset (rho: +0.54; uncorrected p-value: 2.5 × 10^−5^; t-value: +4.6). Figure 2C presents the spatial characteristics of functional coactivation 60 ms after stimulus offset. At this time, functional coactivation was observed through multiple intra-hemispheric fasciculi in the left hemisphere, including local U-fibers (33/138 ROI pairs: 23.9%), arcuate (16/21: 76.2%), IFOF (17/63: 27.0%), SLF (9/20: 45.0%), ILF (7/21: 33.3%), extreme capsule (4/16: 25.0%), MLF (3/11: 27.3%), parietal aslant (3/8: 37.5%), uncinate (5/20: 25.0%), frontal aslant (2/3: 66.7%) and cingulum fasciculus (2/45: 4.4%). The callosal fibers (12/79 ROI pairs: 15.2%) also showed functional coactivation.

### 3.8. Functional coactivation associated with stimulation-induced expressive aphasia

A significant association was likewise noted between increased probability of stimulation-induced expressive aphasia and increased functional coactivation intensity during a period between stimulus offset and response onset (Figure 4C), and the strength of association was maximized at 380 ms before response onset (rho: +0.78; uncorrected p-value: 3.9 × 10^−12^; t-value: +9.0). Figure 2D presents the spatial characteristics of functional coactivation 380 ms before response onset. At this time, functional coactivation was observed through multiple intra-hemispheric fasciculi in the left hemisphere, including local U-fibers (31/138 ROI pairs: 22.5%), arcuate (13/21: 61.9%), IFOF (16/63: 25.4%), extreme capsule (6/16: 37.5%), cingulum (6/45:13.3%), SLF (5/20: 25.0%), ILF (6/21: 28.6%), frontal aslant (3/3: 100%), and uncinate fasciculus (3/20: 15%). The callosal fibers (7/79 ROI pairs: 8.9%) also showed functional coactivation.

### 3.9. Functional coactivation associated with stimulation-induced speech arrest

A significant association was noted between increased probability of stimulation-induced speech arrest and increased functional coactivation intensity around response onset (Figure 4D). Before response, the strength of association was maximized at 50 ms pre-response onset (rho: +0.78; uncorrected p-value: 7.8 × 10^−12^; t-value: +8.8). Figure 2E presents the spatial characteristics of functional coactivation 50 ms before response onset. At this time, functional coactivation was observed through multiple intra-hemispheric fasciculi in the left hemisphere, including local U-fibers (16/138 ROI pairs: 11.6%), extreme capsule (6/16: 37.5%), arcuate (4/21: 19.0%), frontal aslant (3/3: 100%), SLF (3/20: 15.0%), and uncinate fasciculus (2/20: 10.0%). The callosal fibers (8/79 ROI pairs: 10.1%) also showed functional coactivation.

### 3.10. Functional coactivation associated with stimulation-induced face sensorimotor symptoms

A significant association was noted between increased probability of stimulation-induced facial sensorimotor symptoms and increased functional coactivation intensity around response onset, and the strength of association was maximized at 430 ms after response onset (rho: +0.81; uncorrected p-value: 2.0 × 10^−13^; t-value: +9.9; Figure 4E). Figure 2F presents the spatial characteristics of functional coactivation 430 ms after response onset.

### 3.11. Disentangling functional coactivation from high-gamma amplitude effects

As a post hoc analysis, we examined how local high-gamma amplitude and functional coactivation intensity correlated with the probability (%) of five stimulation-induced manifestations during the entire 2,500-ms analysis period. For auditory hallucination, the highest Spearman’s rho was +0.82 with high-gamma and +0.82 with functional coactivation at 220 ms after stimulus onset. For receptive aphasia, it was +0.51 (high-gamma) and +0.54 (functional coactivation) at 60 ms after stimulus offset. For expressive aphasia, it was +0.74 and +0.78, respectively, at 380 ms before response onset. For speech arrest, both correlations were +0.78 at 50 ms before response onset. For face sensorimotor symptoms, they were +0.82 (high-gamma) and +0.81 (functional coactivation) at 430 ms after response onset.

## 4. Discussion

### 4.1. Innovation and significance

For the first time, this study provides a dynamic causal tractography atlas that visualizes millisecond-scale functional coactivation and co-inactivation through specific fasciculi in both hemispheres, clarifying the causal roles of 12 major fasciculi during auditory descriptive naming. In other words, it visualizes the mesoscale spatiotemporal dynamics of neural coordination across white matter pathways within distributed cortical networks essential for understanding and responding to spoken questions. We identified these causal roles by correlating the intensity of functional coactivation with the probability of speech-related clinical manifestations induced by electrical stimulation at the cortical sites where these fasciculi originate. The study revealed the sequential and alternating engagement of fasciculi with causal roles in auditory descriptive naming. No single fasciculus was consistently engaged throughout the task; rather, each fasciculus was active only during specific stages, including auditory perception, semantic-syntactic analysis, lexical retrieval, speech planning/initiation, and sensorimotor processes for articulation. Functional coactivation effects on electrical stimulation findings cannot be attributed solely to cortical high-gamma amplitudes. We found that incorporating functional coactivation through direct white matter pathways increased correlations with receptive and expressive aphasia by 5–6%, yet did not enhance correlations with sensory or motor manifestations. This observation may be consistent with the notion that semantic and syntactic processing, as well as lexical retrieval, rely on extensively distributed networks, whereas sensorimotor functions are mediated by more localized cortical structures.

The present study demonstrated that multiple distinct major fasciculi contribute simultaneously to each of these stages. Our data provide compelling evidence that functional coactivation at discrete time windows is associated with distinct language or speech-related functions, as confirmed by electrical stimulation mapping. Quantitative analysis further supported our hypothesis that the left arcuate fasciculus exhibited the earliest, strongest, and sustained functional coactivation. We also found that functional coactivation was highly left-hemispheric dominant several hundred milliseconds before the response, yet this left-hemispheric dominance appeared milder in left-handed individuals. Collectively, the evidence presented in this study advances current neurobiological models of speech network organization.

The robustness of our findings is supported by analyzing naming-related high-gamma amplitudes from 7,792 intracranial electrode sites across 106 patients, which, to our knowledge, represents the largest sample size reported to date. White matter streamlines were delineated using high-quality DWI tractography data from 1,065 healthy individuals in the HCP dataset (Yeh et al., 2018). We enhanced the generalizability of our atlas by clarifying the impacts of age, handedness, and epilepsy-related profiles on task-related high-gamma modulations. Below, we discuss the significance of functional coactivation in major fasciculi as well as intra-hemispheric U-fibers not classified among the 12 major fasciculi.

### 4.2. Arcuate fasciculus

As shown in Figure 5, the arcuate fasciculus contributes to auditory descriptive naming for the longest periods among the 12 major fasciculi studied in the current study. The arcuate fasciculus was defined as a dorsal fiber bundle connecting the temporal lobe to the frontal or parietal lobe (Supplementary Figure 3B) (Thiebaut de Schotten et al., 2011; Dick and Tremblay, 2012; Hansen et al., 2021; Yeh, 2022). Upon hearing auditory questions, the bilateral arcuate fasciculi between the STG and precentral gyri exhibited functional coactivation. Up to 10–15% of the arcuate pathways in each hemisphere demonstrated functional coactivation (Figure 5). These specific portions of the arcuate fasciculi comprises an articulatory/phonological loop, and the bilateral functional coactivation is attributed to auditory-motor transformation and short-term storage of speech stimuli via subvocal rehearsal (Baddeley, 2003; Warren et al., 2005; Bernal and Ardila, 2009; Cogan et al., 2014; Kambara et al., 2017; Nishida et al., 2017). Auditory-motor transformation refers to the conversion of sensory representations of sound into motor representations, a process suggested to be essential for speech repetition (Warren et al., 2005; Cogan et al., 2014). Behavioral observations from MRI-visible lesions, electrical stimulation, and hemodynamic responses on fMRI suggest that the left arcuate fasciculus terminating at the precentral gyrus is crucial for the short-term storage and repetition of spoken words without requiring lexical retrieval (Bernal and Ardila, 2009; Janssen et al., 2023; Zhao et al., 2023). For example, lesions in the arcuate fasciculus connecting the left STG and precentral gyrus frequently result in speech repetition impairments (Catani et al., 2005; Bernal and Ardila, 2009). Our prior iEEG studies demonstrated that a task requiring sound repetition elicited high-gamma augmentation in the bilateral STG and precentral gyri simultaneously, and that single-pulse electrical stimulation of these high-gamma sites revealed direct connections through the arcuate fasciculi (Nishida et al., 2017; Sonoda et al., 2021). We also previously reported that high-gamma augmentation in the precentral gyri during the working memory maintenance period increased with memory load, indicating the involvement of the precentral gyri in the short-term storage of speech stimuli via subvocal rehearsal (Kambara et al., 2017). The bilateral functional coactivation of the arcuate fasciculi observed in this study is consistent with the notion that auditory-motor transformation is bilaterally distributed (Warren et al., 2005; Cogan et al., 2014). This bilateral distribution supports the ability to continue performing speech repetition even after lexical retrieval impairments following acute lesions in the left arcuate fasciculus (Shuren et al., 1995; Berthier et al., 2012).

Between the question offset and 300 ms before response onset, functional coactivation in the left arcuate fasciculus was extensive, whereas coactivation in the right arcuate fasciculus was less pronounced (Figure 5). During this period, functional coactivation was observed in 60–90% of the left arcuate pathways, compared to only 0–15% of the right. Functional coactivation pathways in the left arcuate fasciculus originated mainly from the STG, middle temporal gyrus (MTG), and inferior temporal gyrus (ITG), extending to the precentral gyrus, posterior inferior frontal gyrus (pIFG), and posterior middle frontal gyrus (pMFG). The probability of stimulation-induced receptive and expressive aphasia was best correlated with the intensity of functional coactivation at 60 ms after stimulus offset (Figure 2C) and 380 ms before response onset (Figure 2D), respectively. In other words, the functional coactivation shown in these figures likely reflect coordinated neural activity required for comprehending questions and generating relevant responses; among all pathways studied, the functional coactivation through the left arcuate fasciculus was the most prominent component.

These findings support the notion that the left arcuate fasciculus plays a dominant role in semantic-syntactic analysis and lexical retrieval. Our observations align with lesion studies, which show that damage to the left, but not the right, arcuate fasciculus leads to significant impairments in these cognitive processes (Bernal and Ardila, 2009; Marchina et al., 2011; Wilson et al., 2011; Fridriksson et al., 2013; Griffiths et al., 2013; Gajardo-Vidal et al., 2021; Giampiccolo and Duffau, 2022). A study of 134 stroke survivors demonstrated that persistent impairments in semantic analysis and lexical retrieval were causally associated with damage to the anterior portion of the left arcuate fasciculus rather than the overlying pIFG (Gajardo-Vidal et al., 2021). Additionally, investigators have reported that electrical stimulation of the left arcuate fasciculus transiently impairs semantic analysis and lexical retrieval (Duffau et al., 2002), and that stimulation of the IFG, connected to the temporal lobe neocortex via the arcuate fasciculus, transiently impairs syntactic analysis (Riva et al., 2022).

Neither the left nor right arcuate fasciculus exhibited functional coactivation 80- to 130-ms prior to response onset (Figure 5). The intensity of functional coactivation during this critical time was closely correlated with the probability of stimulation-induced speech arrest (Figure 4D), with the left extreme capsule, SLF and frontal aslant fasciculus among the white matter pathways showing functional coactivation. These findings suggest that the arcuate fasciculus plays a minimal or modest role in the planning and initiation of overt responses. An alternative interpretation is that the human brain engages in response planning and initiation only after the left arcuate fasciculus completes semantic-syntactic analysis and lexical retrieval. We could not find any prior studies that have distinctly separated the impact of arcuate fasciculus lesions on response initiation from their effects on semantic-syntactic processing or lexical retrieval.

After the onset of overt response, 10–20% of the bilateral arcuate pathways, including those connecting the STG and Rolandic areas, exhibited functional coactivation (Figure 5). A plausible explanation for this observation is that the arcuate fasciculus plays a role in monitoring one’s own voice and making responses with appropriate vocal tone (Zheng et al., 2010; Chang et al., 2013; Greenlee et al., 2013). A prior study reported that an acute lesion involving the right arcuate and SLF resulted in impaired prosody perception (Sammler et al., 2018). Another interpretation is that the functional coactivation during this period reflects the activation of the articulatory/phonological loop through the arcuate fasciculi for the short-term storage of one’s own speech (Baddeley, 2003; Kambara et al., 2017).

### 4.3. Superior longitudinal fasciculus (SLF)

The SLF dorsally connects the parietal-occipital regions and the frontal lobe (Supplementary Figure 3C) (Catani et al., 2005; Dick and Tremblay, 2012). The functional coactivation in the left SLF was extensive and intensified between 300 ms before stimulus offset and response onset (Figure 5), while the right SLF showed functional co-inactivation (Supplementary Figure 5C). Specifically, during this period, up to 15–45% of the left SLF pathways showed functional coactivation, whereas up to 10% of the right SLF pathways showed functional co-inactivation. Given the correlation between functional coactivation intensity and probability of stimulation-induced receptive aphasia, expressive aphasia and speech arrest (Figure 4), the left SLF appears to play a role in semantic-syntactic analysis, lexical retrieval, and response initiation. Previous studies in patients undergoing tumor surgery have reported that surgical damage involving the left SLF and ILF was associated with postoperative impairments in lexical retrieval (Herbet et al., 2016; Tomasino et al., 2018). A study of 46 chronic post-stroke patients found that damaged fasciculi associated with impaired semantic processing included the left ILF as well as the left posterior cortex overlying the SLF (Alyahya et al., 2020). Additionally, a study involving 11 patients with brain tumors reported that electrical stimulation of the anterior-inferior portion of the left SLF resulted in speech arrest (Maldonado et al., 2011).

### 4.4. Middle longitudinal fasciculus (MLF) and inferior longitudinal fasciculus (ILF)

The temporal dynamics of functional coactivation were similar between the left MLF and ILF. Between 300 ms before and after stimulus offset, 25–40% of these fasciculi demonstrated functional coactivation, with the intensity and extent gradually diminishing afterward (Figure 5). Given the association between increased functional coactivation intensity and increased probability of stimulation-induced receptive aphasia (Figure 4), and considering that receptive aphasia can result from impairments in phonological or semantic analysis, the functional coactivation observed in the left MLF and ILF likely play roles in these processes.

The MLF is a ventral fiber bundle connecting the parietal or occipital lobe to the STG (Supplementary Figure 3D). The functional role of the left MLF has been previously understudied. A DWI tractography study involving 29 healthy adult individuals reported that the integrity of the MLF was correlated with phoneme discrimination performance (Tremblay et al., 2019). Another DWI tractography study of 20 patients with primary progressive aphasia reported that the loss of integrity of the left MLF was correlated with impairment in word comprehension and retrieval but not with articulation or fluency (Luo et al., 2020). A study of eight patients with brain tumors reported that picture-naming errors induced by stimulation and resection were attributed to the effects of the left IFOF, but not the left MLF (De Witt Hamer et al., 2011). A future study differentiating the patterns of functional coactivation between auditory and picture naming tasks may provide insights into the functional role of the left MLF.

The ILF ventrally connects the occipital or parietal lobe to the temporal lobe (Supplementary Figure 3E). The functional coactivation dynamics in the left ILF are consistent with the notion that the left ILF contributes to semantic analysis, as indicated by previous studies. A DWI tractography study demonstrated that the anatomical integrity of the left ILF was impaired in 11 children with object recognition impairments compared to age- and sex-matched typically developing children (Ortibus et al., 2012). Additionally, a study of 58 patients with brain tumors reported an association between lesions involving the left ILF and poor performance in a famous face naming task (Burkhardt et al., 2023). Another study of 12 brain tumor patients found that electrical stimulation of the left ILF did not induce language-related manifestations, but a subset of patients developed transient naming errors following resection involving the left ILF (Mandonnet et al., 2007).

### 4.5. Inferior fronto-occipital fasciculus (IFOF)

The IFOF ventrally connects the occipital-posterior temporal regions to the frontal lobe through the external capsule (Supplementary Figure 3F). Functional coactivation in the left IFOF began prior to stimulus offset, peaked approximately 300 ms after stimulus offset, and persisted until 240 ms before response onset (Figure 5). At 300 ms after stimulus offset, 35% of the left IFOF pathways showed functional coactivation. Given the correlation between functional coactivation intensity and probability of stimulation-induced expressive and receptive aphasia (Figure 4), the left IFOF appears to play roles in both lexical retrieval and semantic analysis. A study of 15 stroke survivors reported that lesions in the left IFOF are associated with impaired semantic analysis (Harvey and Schnur, 2015). A study of 31 patients with brain tumor reported that infiltration to the left IFOF was associated with impaired semantic analysis (Zhao et al., 2023). Another study of 12 patients with brain tumor reported that electrical stimulation of the left IFOF elicited anomia (Mandonnet et al., 2007).

### 4.6. Frontal aslant fasciculus

The frontal aslant fasciculus connects the IFG and the superior frontal gyrus (SFG) while running immediately medial to the anterior SLF (Supplementary Figure 3G). Functional coactivation in the left frontal aslant fasciculus was observed after stimulus offset and sustained until overt responses (Figure 5). All three frontal aslant pathways demonstrated functional coactivation at 380 ms and 50 ms before response onset, when coactivation intensity best correlated with probability of stimulation-induced expressive aphasia and speech arrest, respectively (Figure 4). These observations suggest that the left frontal aslant fasciculus may play a causal role in transferring the retrieved word to speech preparation and initiation. Electrical stimulation and lesions involving the left frontal aslant fasciculus have been reported to result in speech arrest and impairments in movement initiation (Vassal and Boutet, 2014; Kinoshita et al., 2015; Chernoff et al., 2018).

### 4.7. Parietal aslant fasciculus

The parietal aslant fasciculus is a fiber bundle coursing laterally to the lateral ventricle, connecting the parietal lobe to the temporal lobe (Supplementary Figure 3H). Functional coactivation was observed in up to 50% of the left parietal aslant pathways during the 600-ms period around stimulus offset, with intensity peaking at 150–190 ms after stimulus offset (Figure 5). Given the strong correlation between functional coactivation intensity and probability of stimulation-induced receptive aphasia (Figure 4), the left parietal aslant fasciculus appears to play a role in semantic comprehension of questions. A previous case study reported that intraoperative electrical stimulation of the left parietal aslant fasciculus elicited semantic paraphasias (Chidambaram et al., 2023).

During the 400-ms period before response onset, up to 45% of the right parietal aslant pathways exhibited functional co-inactivation (Supplementary Figure 5D). Additionally, left-handedness was associated with a milder functional co-inactivation within the right parietal aslant fasciculus. The causal role of this fasciculus in naming has not been previously clarified. One possible explanation for our observations is that the right parietal aslant fasciculus is not essential for lexical retrieval; thus, its neural coordination decreases to reallocate resources to left-hemispheric networks, particularly in right-handed individuals.

### 4.8. Uncinate fasciculus

The uncinate fasciculus is a fiber bundle connecting the anterior portion of the temporal lobe to the ventral portion of the frontal lobe through the temporal stem (Supplementary Figure 3I). Between 200 ms before stimulus offset and 150 ms before response onset, 10–50% of the left uncinate fasciculus exhibited functional coactivation (Figure 5). Given the observed correlation between functional coactivation intensity and probability of stimulation-induced responses (Figure 4), the left uncinate fasciculus likely contributes to semantic analysis and lexical retrieval. This fasciculus remains one of the least understood among the major fasciculi. Investigators infer that the left uncinate fasciculus plays a role in semantic and syntactic analyses (Friederici and Gierhan, 2013; Alyahya et al., 2020). A study of 10 patients with left-hemispheric stroke reported that impairment in semantic control was correlated with reduced resting-state functional connectivity on fMRI and decreased anatomical integrity of the uncinate fasciculus on DWI tractography (Harvey et al., 2013). Conversely, a study of 13 patients with brain tumors reported that neither electrical stimulation nor resection of the left uncinate fasciculus significantly impaired speech processes (Duffau et al., 2009).

### 4.9. Cingulum fasciculus

The cingulum fasciculus is a fiber bundle coursing medially to the lateral ventricle, connecting the occipital, parietal, or temporal lobe to the frontal lobe (Supplementary Figure 3J). Between 400 ms after stimulus offset and 200 ms before response onset, 10–20% of the left cingulum pathways showed significant functional coactivation (Figure 5). Given the correlation between functional coactivation intensity and probability of stimulation-induced manifestations (Figure 4), the left cingulum fasciculus appears to play a role in retrieval of words. A study of 29 patients with drug-resistant focal epilepsy demonstrated that naming errors were elicited by electrical stimulation of the left medial frontal and parietal regions via depth electrodes positioned around the cingulum fasciculus (Perrone-Bertolotti et al., 2020). Additionally, a study of five patients with intractable pain found that stimulation of the left, but not the right, cingulum impaired short-term recall of visual objects (Fedio and Ommaya, 1970).

### 4.10. Extreme capsule

The extreme capsule was defined as a fiber bundle connecting the precentral, postcentral, or paracentral gyrus to the IFG or insular gyrus through the external capsule (Supplementary Figure 3K) (Yeh et al., 2018). Between stimulus offset and response onset, as well as during overt articulation, 25–50% of the left extreme capsule exhibited functional coactivation, with peak intensity occurring immediately prior to response onset. The observed correlation between functional coactivation intensity and probability of stimulation-induced symptoms (Figure 4) suggests that the left extreme capsule may play a causal role in word retrieval and speech initiation. A study of 123 patients with left hemispheric stroke showed that lesions in the left extreme capsule was an independent predictor of aphasia, defined by the Toke Test (Martinez Oeckel et al., 2021). A study of 38 patients with brain tumor reported that intraoperative stimulation of the left extreme capsule elicited picture naming error (Papagno et al., 2011).

### 4.11. Intra-hemispheric U-fibers

Intra-hemispheric U-fibers, defined as those outside the 12 major fasciculi, were extensively distributed (Supplementary Figure 3L). Between stimulus offset and response onset, more than 10% of these U-fibers in the left hemisphere exhibited functional coactivation (Figure 5). This observation suggests that, rather than consistent enhancement of the same U-fiber, specific pairs of neighboring cortices were transiently engaged at different locations and time windows, facilitating local coordination at distinct stages of the naming process.

Key observations reveal that functional coactivation via local U-fibers involving the left IFG or precentral gyrus was enhanced 400 ms after question offset and subsided after response onset (Figure 2D). Given the correlation between functional coactivation intensity and the probabilities of stimulation-induced expressive aphasia and speech arrest (Figure 4), this coactivation likely reflects lexical retrieval and speech planning following the semantic-syntactic analysis of questions. An fMRI study in 26 healthy individuals reported that increased hemodynamic activation in the pars triangularis of the left IFG was linked to semantic processing, while activation in the left precentral gyrus and pars opercularis of the left IFG was related to phonological processing. (Katzev et al., 2013) A study of 134 stroke survivors demonstrated that long-term impairments in lexical retrieval were more strongly associated with damage to the anterior portion of the left arcuate fasciculus than with damage to the left IFG (Gajardo-Vidal et al., 2021).

Additionally, transient functional co-inactivation mediated by local U-fibers in the left IFG was observed during the question listening phase (Figure 2B). This co-inactivation suggests a suppression of neural resources required for lexical retrieval in response to hearing *wh-*questions. Such suppression may optimize neural resource allocation (Norman, 1975), enhancing auditory processing by reducing cognitive load in the left IFG and its associated white matter tracts. This prioritization may facilitate the perception of question stimuli within the STG and connected fasciculi.

### 4.12. Callosal fibers

Callosal fibers connect the left and right hemispheres via the corpus callosum (Supplementary Figure 3M). Inter-hemispheric functional coactivation through these fibers was observed throughout the task (Figure 5), with different portions of the callosal fibers engaged at various stages of the naming process. Upon hearing the questions, functional coactivation was prominent in the posterior portion of the corpus callosum (Figure 2A). In contrast, prior to and during overt responses, functional coactivation was more pronounced in the anterior corpus callosum (Figure 2F). A possible explanation for the inter-hemispheric functional coactivation during stimulus listening is that it integrates neural representations of temporal speech features, such as the timing and rhythm of speech sounds, processed by the left STG, (Zatorre and Belin, 2001; Chang et al., 2010; Floegel et al., 2020) and spectral features, including tone and pitch, processed by the right STG (Zatorre and Belin, 2001; Floegel et al., 2020). One possible explanation for the inter-hemispheric functional coactivation during overt responses is that it supports the coordination of symmetric and synchronous movements of the mouth structures, which are essential for speech initiation and overt articulation (Hoptman and Davidson, 1994; Hervé et al., 2013). The causal role of the corpus callosum in initiating speech is highlighted by reports of mutism immediately after callosotomy (Sussman et al., 1983; Crutchfield et al., 1994).

### 4.13. Anterior commissural fibers

The anterior commissural fibers are those connecting the left and right temporal lobes through the anterior commissure (Supplementary Figure 3N). In the present study, only one connecting between the left and right ITG was found in our tractography analysis. We failed to observe functional coactivation or co-inactivation through the anterior commissural fibers (Figure 5).

### 4.14. Impacts of patient demographics on neural measures

In our study cohort, older patients exhibited shorter response times compared to younger patients. Specifically, each additional √year (e.g., from 9 to 16 years) was associated with a 246-ms reduction in response time. Additionally, a shorter response time was associated with greater high-gamma augmentation in the left precentral gyrus between 200–500 ms after question offset. For every one-second reduction in response time, there was an 8–9% increase in high-gamma amplitude in the left precentral gyrus during this window. These findings suggest that individuals with shorter response times show greater neural engagement in the left precentral gyrus 200–500 ms after question offset, whereas older individuals tend to respond more quickly. Therefore, caution is warranted when interpreting the dynamic tractography atlas 200–500 ms after question offset. We did not observe a significant effect of response time on neural measures time-locked to question onset or response onset.

We also found that left-handedness was linked to higher high-gamma amplitude (i.e., less degree of high-gamma attenuation) in two right-hemispheric ROIs 200–500 ms before response onset. Specifically, compared to right-handed individuals, left-handed individuals exhibited high-gamma amplitudes higher in the right entorhinal cortex and right supramarginal gyrus by 9–14%. This finding suggests that left-hemispheric dominant neural coordination during the lexical retrieval period is enhanced in right-handed individuals, whereas left-handed individuals show a less pronounced left-hemispheric dominance. Our observation is consistent with fMRI studies reporting that left-handed individuals, compared to right-handed ones, less frequently exhibit left-hemispheric dominant hemodynamic activation during language tasks. (Szaflarski et al., 2002; Mazoyer et al., 2014) Aside from handedness, we did not find any other patient demographics or epilepsy-related profiles associated with high-gamma measures in the present study.

### 4.15. Methodological considerations

In the present study, we define pairs of jointly activated brain regions directly connected by white matter as a ‘functional coactivation network’. We intentionally avoid using the term ‘functional connectivity network’, as iEEG researchers often define functional connectivity based on temporal coupling evaluated through trial-by-trial analysis of iEEG signals within individual patients (Bettus et al., 2011; Kucyi et al., 2018). In contrast, we characterize the spatiotemporal dynamics of group-level high-gamma amplitude modulations using iEEG data combined from different subsets of patients (Kitazawa et al., 2023; 2025). Our analytical approach closely parallels meta-analyses of fMRI studies, which integrate data from multiple participant cohorts to identify brain coactivation networks (i.e., cortical regions jointly activated during cognitive or sensorimotor tasks) (Crossley et al., 2013). Notably, Crossley et al. (2013) demonstrated in 27 healthy volunteers that resting-state functional connectivity networks derived from individual-level analyses exhibited community structures spatially consistent with those obtained via meta-analytic coactivation methods. Below, we provide a detailed rationale, justification, validation, and discussion of limitations regarding our brain coactivation network analysis.

Because electrode placement in iEEG is strictly guided by clinical necessity, comprehensive bilateral sampling of nonepileptic homotopic regions is ethically and practically infeasible. Consequently, individual-level analyses alone do not fully characterize interhemispheric connectivity or coactivation dynamics between these regions, despite evidence that homotopic cortical areas communicate directly through inter-hemispheric white matter fibers. For instance, previous iEEG studies have demonstrated that frontal cortices can transmit neural information directly to homotopic regions in the opposite hemisphere via the corpus callosum within approximately 30 milliseconds, as evidenced by cortical response latencies following single-pulse electrical stimulation (Trebaul et al., 2018; Mitsuhashi et al., 2021). Additionally, studies of healthy adults have shown that transcranial magnetic stimulation applied to the motor cortex can result in either facilitation or inhibition of activity in the contralateral motor cortex (Ferbert et al., 1992; Hanajima et al., 2001; Daskalakis et al., 2002; Bortoletto et al., 2021).

In light of spatial constraints inherent to iEEG signal sampling, investigators have employed a “virtual brain” approach, integrating data from multiple patients into a standardized brain atlas and estimating region-to-region neural interactions using summary iEEG measures derived from distinct patient subsets (Kunii et al., 2013; Burke et al., 2014; Avanzini et al., 2016; Kitazawa et al., 2025). This method is analogous to a cross-sectional study design, wherein population-level time-related effects are inferred from data across different participants rather than repeated longitudinal measures from the same individuals. For example, a prior iEEG study documented normative developmental changes in iEEG measures using cross-sectional data because obtaining long-term, repeated iEEG recordings from non-epileptic regions within the same participants is impractical (Sakakura et al., 2023). As cross-sectional designs are methodologically weaker than longitudinal ones, our group-level, iEEG-based co-activation network analysis has inherent limitations compared with individual-level iEEG connectivity studies (Bettus et al., 2011; Kucyi et al., 2018). The chief limitation is the additional inference—within the Step 1 analysis outlined below—required to generalize group-level high-gamma amplitude dynamics observed in specific ROIs to the broader patient population. Nevertheless, supporting evidence in Supplementary Table 3 and Supplementary Figures 6–7 suggests such generalization is plausible. A notable strength of our group-level co-activation analysis is that Step 3 draws on a large patient cohort, enhancing generalizability.

Many previously-reported iEEG-based connectivity studies followed three main stages:

[Step 1] – Identification of between-site temporal coupling. Detect temporal coupling between electrode sites using iEEG dynamics; functional connectivity is inferred when statistical analyses reveal synchronous or coherent patterns, or predictive modulation.

[Step 2] – Assessment of mechanistic relevance. Assess the mechanistic relevance of these couplings by correlating them with independent neuroimaging, electrophysiological, or behavioral measures.

[Step 3] – Generalization of observed findings. Extrapolate patient-specific connectivity patterns to a broader clinical population.

For instance, a study of 5 patients with drug-resistant focal epilepsy aimed to characterize functional connectivity patterns between distinct lobes within three intrinsic brain networks in the same hemisphere (Kucyi et al., 2018). Specifically, the investigators focused on the default (medial prefrontal and posteromedial cortex), dorsal attention (frontal eye fields and superior parietal lobule), and frontoparietal control (inferior parietal lobule and dorsolateral prefrontal cortex) networks. Electrodes were placed within these networks in varying combinations across patients: 3 patients had electrodes in the default network, 2 patients in the dorsal attention network, and 2 patients in the frontoparietal control network.

[Step 1] Kucyi et al., (2018) employed Pearson correlation analysis to identify electrode pairs exhibiting coherent high-frequency band (70–170 Hz) envelope fluctuations during resting periods. Based on statistical evidence, the investigators inferred the existence of robust iEEG-based functional connectivity within each intrinsic network of interest in each patient. The degree of temporal coupling within the default, dorsal attention, and frontoparietal networks was reported as Pearson *r*-values of 0.77, 0.36, and 0.46 in a patient.

[Step 2] They found spatial correlations between iEEG-based and fMRI-based functional connectivity measures within each patient (Pearson *r*-values ranging from 0.38 to 0.61).

[Step 3] Kucyi et al., (2018) concluded that intrinsic network activity patterns showed strong similarities between fMRI and iEEG recordings from the same patients. Furthermore, they inferred that electrophysiologically-defined intrinsic functional connectivity is likely generalizable across broader individuals, brain networks, and behavioral states.

Another study of 5 patients with drug-resistant temporal lobe epilepsy aimed to characterize functional connectivity patterns in cortical areas affected and unaffected by interictal epileptiform discharges using iEEG and fMRI (Bettus et al., 2011).

[Step 1] Bettus et al., (2011) performed nonlinear regression analysis to identify electrode pairs exhibiting coherent fluctuations in iEEG signals during resting-state periods. They inferred iEEG-based functional connectivity between electrode pairs based on statistical evidence (h^2^ values range: 0.1 to 0.2).

[Step 2] They found that functional connectivity, as measured by iEEG, was higher in areas affected by interictal epileptiform discharges compared to unaffected areas. Conversely, fMRI findings indicated greater connectivity in areas unaffected by epileptiform discharges.

[Step 3] Bettus et al., (2011) concluded that functional connectivity measures derived from iEEG and fMRI provide complementary but sometimes inconsistent information in drug-resistant temporal lobe epilepsy.

In our iEEG study of 106 patients with drug-resistant focal epilepsy, we aimed to characterize the dynamic patterns of functional coactivation network across 54 ROIs. The aforementioned iEEG studies (Bettus et al., 2011; Kucyi et al., 2018) were designed to determine functional connectivity strength averaged over extended resting periods, validating or correlating their iEEG-derived connectivity with complementary fMRI findings. In contrast, our iEEG study was designed to capture rapid functional coactivation dynamics on the order of tens of milliseconds. We enhanced the biological plausibility of our reported coactivation patterns using MRI tractography and electrical stimulation mapping. By explicitly detailing each analytic step, we clarify how our definition of functional coactivation differs from that of functional connectivity used in previous iEEG studies (Bettus et al., 2011; Kucyi et al., 2018), and acknowledge limitations specific to our approach.

[Step 1] We performed a one-sample t-test to identify the timing of significant high-gamma augmentation at each ROI. The null hypothesis stated that the group-level high-gamma amplitude at each time bin did not differ significantly from zero. As detailed in the Methods section above, significant high-gamma augmentation was defined using a 99.99% CI and a minimum required duration of 50 ms. Even under a scenario involving extreme autocorrelation of high-gamma amplitude augmentation, the probability of observing significant high-gamma augmentation by chance at each ROI was very low (2.46 × 10^−6^).

Here, we acknowledge a limitation regarding generalizability when interpreting significant high-gamma augmentation observed at the group level within each ROI. Despite the excellent fidelity of iEEG signals—typically more than 100-fold greater than scalp EEG (Ball et al., 2009)—we recognize substantial inter-patient variability in high-gamma amplitudes. Consequently, pairing data from an ROI in one patient with the homotopic ROI in another could artificially inflate functional coactivation estimates due to spurious correlations. This inherent across-patient variability underscores the necessity of a group-level analysis to reliably approximate the population-level temporal dynamics of high-gamma activity. To assess the robustness and representativeness of our observed temporal profiles of mean high-gamma activity within a given ROI, we conducted additional analyses using a leave-one-out approach (Supplementary Table 3, Supplementary Figure 6). Spearman’s correlation coefficients (rho) demonstrated minimal impact following the exclusion of any single patient, with mean rho values across 54 ROIs ranging from 0.946 to 0.999 (p-values: 1.4 × 10^−189^ to 1.8 × 10^−5^), confirming the robustness of the observed high-gamma dynamics at each ROI. This finding indicates that the detected high-gamma modulation reflects a stable group-level pattern rather than being driven by an exceptional individual. Supplementary Figure 7 illustrates temporal changes in mean high-gamma amplitude at each ROI, with 95% CI bars that are 1.96 times wider than conventional standard error bars. Readers may notice that the 95% CI bars became substantially narrower as the number of contributing patients increased. Collectively, these findings support our inference that significant high-gamma augmentation observed at the group level within each ROI can reasonably be generalized to a broader patient population.

We acknowledge that our virtual brain framework emphasized group-level trends of high-gamma dynamics and did not capture individual-level patterns. Additionally, cohort-specific effects related to patient demographics and epilepsy characteristics should be considered. To account for inter-individual differences in behavior and neural dynamics, we thoroughly evaluated potential confounding factors, specifically considering patient age, handedness, epileptogenic hemisphere, MRI-visible lesions, antiseizure polytherapy, and response time. A higher number of antiseizure medications indicates a greater seizure-related cognitive burden (Kwan and Brodie, 2001). Response times also serve as a cognitive performance measure for the naming task. Thus, our analytic approach accounts for the effect of a patient’s cognitive skill on neural measures. Including too many covariates in the mixed model analysis could introduce collinearity issues. In the Supplementary Document, we provide the results of ancillary analyses for interested readers, examining (1) the relationship between a history of bilateral tonic–clonic seizures and task behaviors, and (2) the relationship between patient age and the stimulus intensity required to induce clinical manifestations.

To characterize the functional coactivation network, we identified periods during which pairs of ROIs exhibited simultaneous high-gamma amplitude augmentation. Functional coactivation between two ROIs was defined using two criteria: (i) both ROIs must demonstrate significant, simultaneous high-gamma augmentation persisting for at least five consecutive 10-ms time bins, and (ii) the ROIs must be directly connected by a streamline derived from MRI tractography. A distinctive feature of our approach is the integration of millisecond-scale high-gamma co-augmentation with anatomically confirmed white matter pathways linking cortical ROIs. As detailed in the Methods section, even under a scenario accounting for extreme autocorrelation of high-gamma amplitude augmentation, the probability of observing simultaneous high-gamma co-augmentation by chance was exceedingly low (3.52 × 10^−7^).

By incorporating the presence of direct streamlines connecting these cortical ROIs as a criterion, the current study enhanced the biological plausibility of the observed functional coactivation. It is suggested that cortical regions with simultaneous high-frequency neural responses are involved in coordinated interactions (Singer, 1993; Buonomano and Merzenich, 1998). Our previous iEEG study demonstrated that single-pulse electrical stimulation at a cortical site elicited neural responses within 50 ms specifically at distinct cortical sites showing significant, simultaneous, and sustained naming-related high-gamma augmentation (Sonoda et al., 2021). Collective evidence supports the notion that neural coordination can plausibly occur between these cortical ROIs directly connected by white matter streamlines.

We inferred functional coactivation between pairs of ROIs exhibiting significant, simultaneous, and sustained high-gamma augmentation within our virtual brain framework, based on statistical analysis of iEEG signals and MRI tractography-derived white matter streamlines.

[Step 2] This analysis aimed to elucidate the mechanistic significance of functional coactivation patterns by assessing the relationship between mean coactivation intensity within each time bin and the probability of eliciting specific stimulation-induced symptoms at each ROI. Integration of electrical stimulation mapping is a distinctive aspect of our iEEG analytical approach. Specifically, functional coactivation measured 220–250 ms after auditory stimulus onset (during auditory stimulus listening) showed the strongest association with stimulation-induced auditory hallucination (Spearman’s rho = +0.82; p = 6.6 × 10^−14^), suggesting its role in auditory perceptual processing. Functional coactivation at 60 ms after stimulus offset was strongly correlated with stimulation-induced receptive aphasia (rho = +0.54; p = 2.5 × 10^−5^), implicating its role in semantic-syntactic analysis. Functional coactivation measured 380 ms before response onset was most strongly associated with stimulation-induced expressive aphasia (rho = +0.78; p = 3.9 × 10^−12^), indicating involvement in lexical retrieval. Functional coactivation measured 50 ms before response onset showed a strong association with stimulation-induced speech arrest (rho = +0.78; p = 7.8 × 10^−12^), suggesting a role in speech initiation. Lastly, functional coactivation measured 430 ms after response onset demonstrated the strongest association with stimulation-induced face sensorimotor symptoms (rho = +0.81; p = 2.0 × 10^−13^), supporting its contribution to facial sensorimotor control.

[Step 3] The aforementioned group-level functional coactivation patterns are supported by statistical evidence, align with plausible structural white matter pathways, and have been validated through electrical stimulation mapping. Thus, we infer that these coactivation findings are generalizable to broader patient populations. An advantage of our group-level approach is that it incorporates data from a substantially larger patient cohort than studies relying solely on individual-level connectivity analyses. In our study, dozens of patients contributed data for computing functional coactivation intensity between ROI pairs (Supplementary Table 2). By contrast, studies using only individual-level iEEG-based connectivity analysis often generalize their findings from relatively few patients.

Further discussion regarding the observed spatial and temporal heterogeneity in high-gamma augmentation is warranted. For example, the left STG and posterior IFG exhibited significant high-gamma augmentation for more than 50% of the 2,500-ms analysis period, whereas many ROIs within the right-hemispheric association cortices showed no augmentation at all (Supplementary Figure 7). Additionally, the number of ROIs demonstrating significant high-gamma augmentation immediately after stimulus offset was substantially greater than during the initial 500 ms following stimulus onset (Supplementary Figure 8). Some readers might question whether high-gamma amplitudes observed in these left perisylvian ROIs—particularly immediately after stimulus offset—should have been evaluated using a more conservative threshold to reduce the risk of false-positive detection of task-related high-gamma augmentation. However, to minimize the risk of circular analysis (Kriegeskorte et al., 2009), we uniformly applied the same significance threshold across all ROIs and analysis time bins, without post-hoc adjustment for observed spatial and temporal heterogeneity. As recommended by Kriegeskorte et al. (2009), analytical strategies should be predetermined independently of the observed results.

To define the spatial extent of ROIs, we utilized the Desikan-Killiany atlas, one of the most widely used atlases in neuroscience research (Desikan et al., 2006), and delineated white matter streamlines connecting pairs of these ROIs. Due to the varying sizes of the ROIs, we opted not to perform statistical comparisons of the extent or intensity of functional coactivation between different fasciculi within a given hemisphere, as such comparisons could be misleading. However, we considered it feasible to compare the extent or intensity of functional coactivation within the same fasciculus across different time windows. We acknowledge that our analytical approach resulted in false negatives when delineating genuine streamlines between ROIs. To ensure clarity in our visualizations, we chose to delineate a single white matter streamline for each ROI pair. This approach was necessary to effectively visualize all major fasciculi in bird’s-eye, posterior, and lateral views. Including too many lateral fasciculi would obscure the visualization of medial fasciculi, while an excess of superior fasciculi would hinder the visibility of inferior fasciculi.

We inferred the causal roles of specific fasciculi using clinical manifestations induced by cortical stimulation. Subcortical stimulation, often performed for adult patients with brain tumor during awake craniotomy, was not conducted in our study cohort, as it was infeasible given that most participants were children and the eloquent cortex was identified through standard-care management during extraoperative iEEG monitoring (Nakai et al., 2017; Sonoda et al., 2022).

The present study analyzed high-gamma activity recorded exclusively at the cortical level, allowing us to directly project these measures onto a standard cortical surface space without additional processing required for mapping iEEG data obtained from deep white matter areas. Previous studies have demonstrated that iEEG signals sampled via stereoencephalography-depth electrodes can indeed be projected onto the FreeSurfer cortical surface space (Scorza et al., 2020; Williams et al., 2023). Although subcortical structures were not included in our analysis, stereoencephalography-based sampling from subcortical regions, such as thalamic nuclei, is becoming increasingly common in presurgical evaluations aimed at identifying optimal targets for deep brain stimulation and responsive neurostimulation. Future research is thus warranted to elucidate the functional coactivation and co-inactivation networks involving these subcortical structures. Such studies would likely benefit from employing a volume-based spatial representation (e.g., Ervin et al., 2021; Dubarry et al., 2022; Jaroszynski et al., 2022; Mercier et al., 2022; Qi et al., 2022; Soroush et al., 2023; Williams et al., 2023) in conjunction with the standard cortical surface-based template for visualization.

## Supplementary Material

1

2

Supplementary materials

Supplementary material associated with this article can be found, in the online version, at doi:10.1016/j.neuroimage.2025.121319.

## Data Availability

We have provided iEEG files recorded from all study patients at https://openneuro.org/datasets/ds005545/versions/1.0.3 (doi: 10.18112/openneuro.ds005545.v1.0.3). These publicly available iEEG data have been converted to EDF format, which effectively removes identifiable patient information. Our analyses were conducted using the original Nihon Kohden files, which directly included precise timestamps for stimulus onset, stimulus offset, and response onset, rather than the EDF-converted files. Researchers interested in analyzing our iEEG data are encouraged to contact the corresponding author.

## Abbreviations:

CI: confidence interval
DTI: diffusion tensor imaging
DWI: diffusion-weighted imaging
fMRI: functional magnetic resonance imaging
HCP: Human Connectome Project
IFOF: inferior fronto-occipital fasciculus
ILF: inferior longitudinal fasciculus
ITG: inferior temporal gyrus
iEEG: intracranial electroencephalography
MLF: middle longitudinal fasciculus
MTG: middle temporal gyrus
MNI: Montreal Neurological Institute
pIFG: posterior inferior frontal gyrus
pMFG: posterior middle frontal gyrus
ROI: region of interest
SOZ: seizure onset zone
SFG: superior frontal gyrus
SLF: superior longitudinal fasciculus
STG: superior temporal gyrus
